# Global and Regional Economic Burden of Eating Disorders: A Systematic Review and Critique of Methods

**DOI:** 10.1002/eat.24302

**Published:** 2024-11-14

**Authors:** Moin Ahmed, Md Deen Islam, Phillip Aouad, Jane Miskovic‐Wheatley, Stephen Touyz, Sarah Maguire, Michelle Cunich

**Affiliations:** ^1^ MAINSTREAM The Australian National Centre for Health System Research and Translation Sydney New South Wales Australia; ^2^ Boden Initiative, Charles Perkins Centre, Central Clinical School, Faculty of Medicine and Health The University of Sydney Sydney New South Wales Australia; ^3^ Department of Economics University of Dhaka Dhaka Bangladesh; ^4^ InsideOut Institute for Eating Disorders The University of Sydney and Sydney Local Health District Sydney New South Wales Australia; ^5^ Cardiovascular Initiative, Faculty of Medicine and Health The University of Sydney Sydney New South Wales Australia; ^6^ Sydney Institute for Women, Children and Their Families Sydney New South Wales Australia; ^7^ Sydney Local Health District Sydney New South Wales Australia

**Keywords:** burden of disease, cost of illness, eating disorders, economic cost, health economics

## Abstract

**Objective:**

This systematic review aims to comprehensively examine up‐to‐date evidence on the economic burden of eating disorders (EDs), both globally and by region.

**Methodology:**

A comprehensive search within five electronic databases, MEDLINE, Embase, CINAHL, PsycINFO, and EconLit, retrieved studies published from August 1, 2013, to June 30, 2024. Cost of illness (COI) studies, burden of disease, and other cost studies that reported costs in monetary values were included, and cost‐effectiveness analysis studies were excluded. The quality of COI studies was assessed using Schnitzler's checklist. All cost estimates were converted into 2024 USD purchasing power parity (PPP). The PROSPERO registration number is CRD42022358136.

**Findings:**

Twenty‐six studies were identified for inclusion in this review, with 11 COI studies. The nationwide annual financial cost of EDs is estimated at PPP‐USD 70.5 billion. Indirect costs contributed 70%–93% of total financial costs in the reviewed studies. Intangible costs (burden of disease) were estimated to be PPP‐USD 355.6 billion. About half of the COI studies met 60% of the elements of Schnitzler's checklist, either completely or partly.

**Discussion:**

The number of COI studies has more than doubled in the last 10 years. Findings can inform healthcare administrators/policymakers to understand the magnitude of this burden when setting healthcare priorities and allocating resources to maximize social welfare. However, there are variations in the methods (thus quality) and perspectives used to assess this economic burden. Findings suggest that there is potential for enhancing the methodological rigor of future research.

**Summary**

- This systematic review of the economic costs associated with EDs reveals the significant financial and nonfinancial burden these conditions impose on individuals, healthcare systems, and societies globally.
- The review found that comprehensive economic burden studies are lacking in many countries.
- This review highlighted that it is important to improve the methodological quality of future research in terms of design, analysis, and reporting of results.

## 1 Introduction

Eating disorders (EDs) are serious, complex, psychiatric disorders that lead to significant health and psychosocial complications, along with increased mortality rates (Hambleton et al. 2022; Weigel, Löwe, and Kohlmann 2019). In particular, EDs are a concern for healthcare administrators due to the associated severity of effects on physical and mental health, critical and complex healthcare needs, and high costs to health systems (Simon, Schmidt, and Pilling 2005). Despite the significant personal and economic challenges posed by EDs, healthcare administrators and policymakers have paid relatively little attention to health economic research on EDs, compared to other mental health conditions (Weissman and Rosselli 2017).

In examining the evidence on the cost of EDs using economic analyses, healthcare administrators and policymakers can make informed decisions to address policy gaps regarding the optimum allocation of resources within the health sector and economy (i.e., priorities). In recent times, there has been a growing number of economic studies on EDs (Crow 2014), mainly due to the need to assess costs related to the rise in the prevalence of EDs. These economic studies can be broadly divided into cost of Illness (COI) studies and cost‐effectiveness studies. COI studies aim to quantify the total economic burden of a particular disease or health condition, including direct medical costs, indirect costs such as lost productivity, and intangible costs such as pain and suffering. In contrast, cost‐effectiveness studies compare the relative costs and outcomes of different interventions, assessing which option provides the best value for money (efficiency gains). However, current and comprehensive information on the economic costs associated with EDs, consolidated in a single review, is lacking. This makes the comparison of economic impact of EDs across countries very challenging and limits the understanding of the economic burden of EDs compared across different diseases.

The latest systematic reviews on the economic burden of EDs date back 10 years, when Agh et al. (2015, 2016) conducted two systematic reviews of the literature published between 2009 and mid‐2013 on epidemiology, health‐related quality of life and the economic burden of anorexia nervosa (AN), bulimia nervosa (BN), and binge eating disorders (BED) globally. Earlier, Simon, Schmidt, and Pilling (2005) published a systematic review, including studies from 1980 to 2002, and concluded that the costs associated with EDs were not sufficiently researched. Stuhldreher et al. (2012) systematically reviewed the literature until January 2011 on the economic burden of EDs and concluded that there were limited comprehensive estimates of costs associated with EDs. Although not systematic reviews, other studies include reviews by Weissman and Rosselli (2017) and van Hoeken and Hoek (2020) that discussed cost studies of EDs. These previous studies either considered limited types of eating disorders (Agh et al. 2015, 2016), did not distinguish between COI studies and cost‐effectiveness studies (Agh et al. 2015, 2016), performed limited quality assessments based on an economic checklist (Simon, Schmidt, and Pilling 2005; Weissman and Rosselli 2017; Agh et al. 2015, 2016; van Hoeken and Hoek 2020), did not perform a comprehensive systematic review (Weissman and Rosselli 2017; van Hoeken and Hoek 2020), or did not compare nationwide/regional total annual costs (Stuhldreher et al. 2012).

To our knowledge, no systematic review has comprehensively synthesized and compared evidence on the global economic burden impact of EDs for over a decade. This study aims to undertake a systematic review of the economic burden of EDs that will summarize the latest evidence on the regional (within‐country) and global economic burden, focusing on direct and indirect costs, providing comparable estimates of burden, and critically appraising economic methodologies.

## 2 Methods

This systematic review was undertaken in accordance with Preferred Reporting Items for Systematic Reviews and Meta‐Analyses (PRISMA) guidelines (Page et al. 2021), following a published protocol registration (PROSPERO registration: CRD42022358136). One deviation has been made during the conduct of this systematic review. Schnitzler et al.'s (2023) checklist for COI studies was used instead of the Guide to Critical Evaluation by (Larg and Moss (2011)). Schnitzler et al.'s (2023) checklist was used over two available checklists for the assessment of COI studies, that are the Guide to Critical Evaluation by Larg and Moss (2011), and the Development and Assessment of Cost‐of‐Illness Studies by Müller et al. (2018), as Schnitzler et al. (2023) checklist is developed based on consensus and expert opinion, and not specific to a particular country (Müller et al. (2018) checklist was developed for the German context).

### 2.1 Search Strategy and Database

A comprehensive search from five electronic databases, including MEDLINE, Embase, CINAHL, PsycINFO, and EconLit; was carried out to retrieve the literature on the economic burden of EDs. The search strings and subject headings were developed by MA and MC in consultation with eating disorder expert authors, as well as an experienced academic librarian from the University of Sydney. The detailed search strings are presented in Tables S1–S3. Databases were searched for studies published between August 1, 2013, and June 30, 2024. The search records were exported into EndNote version 20 (The EndNote Team 2013), and duplicates were removed. Duplicate records were further removed in Covidence (Covidence Systematic Review Software n.d.) and Rayyan (Ouzzani et al. 2016) before the commencement of the screening.

### 2.2 Study Selection

Two independent reviewers (M.A. and M.D.I.) independently screened the records in two stages. In the first stage, the abstract and titles of each record were screened in Rayyan. Studies included after the first stage screening were further considered for second stage screening. If there was uncertainty in the eligibility of any record, it was included in the second stage screening. In the second stage of screening, the same reviewers independently made a full assessment of each record after retrieving the full text based on the inclusion and exclusion criteria of this review. Any disagreements were resolved by discussion with a third reviewer (M.C.).

The selection criteria did not limit the language of the publications, minimizing language bias. Google translator was used in case of no English translation provided by the journals. The inclusion and exclusion criteria are listed below.

Inclusion criteria:

- Original research articles and reports published in any language were included.
- Studies that reported COI, healthcare expenditure, or resource utilization for any type of ED were considered.
- Studies that reported economic burden in monetary values (any currency) were included.

Exclusion criteria:

- Cost‐effectiveness studies were excluded.
- Studies that only reported on the costs of a specific intervention were excluded.
- Conference abstracts, review papers, case reports, letters, comments, or editorials were not included.
- Studies that only reported disease burden without monetary values were excluded.

The reason was recorded in Rayyan (and mentioned in PRISMA figure), if a study was excluded. Studies in this review were classified as COI studies and other cost studies (OCS). Included studies were classified as COI studies if the total annual costs of EDs were estimated for a nation or region; otherwise, they were categorized as OCS (Jo 2014).

### 2.3 Defining Cost Measures

**FIGURE 1:**
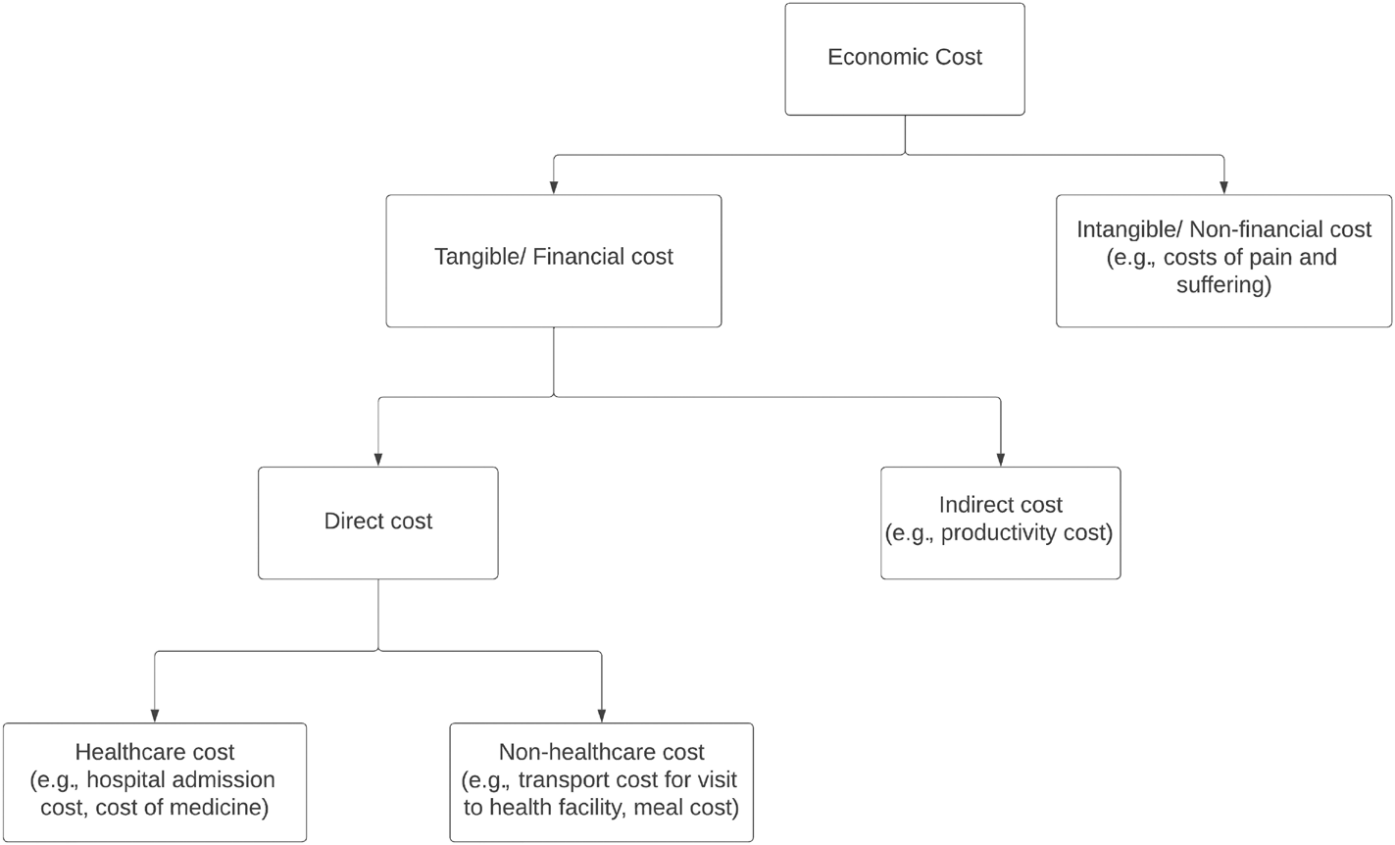
Classification of cost measures.

Cost measures are usually divided into three categories in cost studies‐direct, indirect, and intangible costs (Jo 2014; Koopmanschap 1998; Sarah, David, and James 2000), where direct and indirect costs are classified as tangible costs (Streatfeild et al. 2021; Tannous et al. 2021). The classification of the cost measures is shown in Figure 1.

Direct costs are defined as expenses that are incurred by the healthcare system, society, families, and individual patients, and are further divided into healthcare and nonhealthcare costs (Jo 2014; Tarricone 2006). Healthcare costs encompass expenditures for medical care, such as diagnosis, treatment, and rehabilitation, while nonhealthcare costs include expenses related to nonmedical resources, such as transportation, household expenses, relocation, property losses, and informal care of any kind, associated with the disease (Jo 2014; Tarricone 2006).

Indirect costs, as defined in economic studies, refer to productivity losses due to morbidity and mortality, which are borne by individuals, families, society, or employers (Jo 2014; Koopmanschap 1998; Tarricone 2006). These costs are typically valued using the human capital approach, the friction cost method, or the willingness‐to‐pay approach (Jo 2014; Tarricone 2006).

Intangible costs refer to costs related to pain or psychological suffering (Streatfeild et al. 2021). This loss of well‐being can be measured in terms of disability‐adjusted life years (DALYs) or quality‐adjusted life years (QALYs) (Jo 2014; Streatfeild et al. 2021) and can be combined with the value of statistical life years (VSLY) to estimate the monetary value of the loss attributed to years of disability or premature death associated with EDs (Streatfeild et al. 2021).

### 2.4 Defining Methodological Characteristics

#### 2.4.1 Perspectives of Cost Studies

Cost studies can be conducted from a range of perspectives, such as societal, healthcare system (public third‐party payer), other third‐party payer (health insurance companies), individuals/families, government, and so on. (Jo 2014; Tarricone 2006). Each perspective includes different types of costs, which can result in different cost estimates for the same disease condition. Among these different perspectives, the social perspective is the most comprehensive, which includes all tangible (direct and indirect costs) and intangible costs for all members of the society that are impacted by the disease, whereas the healthcare system perspective only includes direct costs incurred by the healthcare system (Jo 2014; Tarricone 2006).

#### 2.4.2 Epidemiological Approach

Cost studies can be categorized as either prevalence‐based or incidence‐based, depending on how the epidemiological data are utilized. The more common prevalence‐based approach calculates the economic burden of a condition over a specific period, typically 1 year. In contrast, the incidence‐based approach estimates the lifetime costs of a condition, tracking new cases from onset to resolution, either through cure or death, within a defined time period (Jo 2014).

#### 2.4.3 Costing Method

Direct costs can be estimated using different approaches: top‐down, bottom‐up, and using econometric methods (Jo 2014). The top‐down approach allocates aggregate healthcare expenditures to specific diseases or conditions based on overall data, while the bottom‐up approach aggregates micro‐level (usually patient‐level) costs to estimate the total expenditure. The econometric approach, on the other hand, uses econometric (or statistical) methods to estimate the relationship between healthcare costs and disease‐specific factors, allowing for a more nuanced understanding of cost drivers.

Indirect costs can be calculated using the human capital method, the friction cost method and the willingness to pay method (Jo 2014). The human capital method calculates the economic impact of lost labor productivity due to illness or early death by estimating the total earnings lost over an individual's working life, whereas the friction cost method estimates the value of human capital by calculating the present value of a worker's future earnings that is temporarily covered by another person from the unemployment pool until the sick or impaired worker is able to return to work or is permanently replaced. The willingness to pay method quantifies the amount an individual is willing to spend to lower the likelihood of illness or mortality.

#### 2.4.4 Discounting

Discounting is an economic method that reflects an individual's (or society's) preference for receiving income or making payments today rather than in the future (Jo 2014). Discounting is only applicable if costs are estimated over more than 1 year.

### 2.5 Data Extraction, Data Analysis and Quality Assessment

Data were extracted using Microsoft Excel tables. All cost estimates were converted into 2024 USD using gross domestic product (GDP) deflator and purchasing power parity (PPP) exchange rates of relevant countries (International Monetary Fund 2023). Converted cost figures are expressed as PPP‐USD. Tangible costs are reported as direct and indirect costs, and total costs are reported as the sum of direct and indirect costs. In addition, health system costs (a component of direct cost) are also presented. Intangible costs, such as cost of pain and suffering, are reported separately. Stata version 18, R version 2023.12.1 and Microsoft Excel were used to analyze and visualize the data. Quality assessment was performed using Schnitzler et al.'s (2023) consensus‐based checklist for COI studies.

## 3 Results

### 3.1 Search Results

**FIGURE 2:**
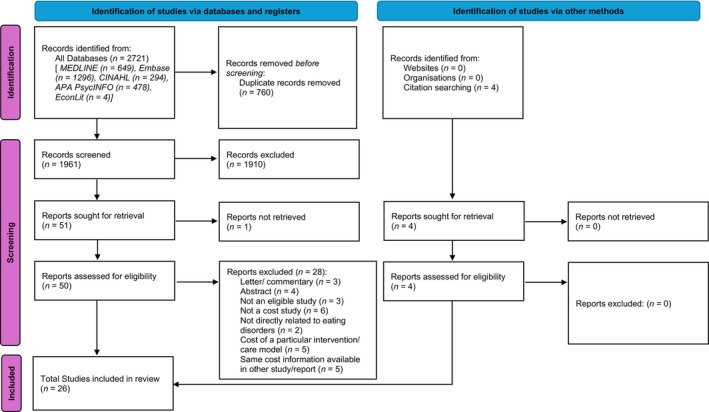
PRIMA 2020 flow diagram for selection of research studies.

A total of 2721 records were retrieved from electronic databases. After removing 760 duplicates, 1961 records underwent title and abstract screening (first‐stage screening). Only one abstract required English translation using Google translator (Table S4). After excluding 1910 records, 50 studies were screened with full text (full text was not found for one study). Some of the economic cost studies were excluded as these literature had identical cost information available in other studies (Table S5). A total of 22 studies met the inclusion criteria (second‐stage screening), and four additional studies were identified from the reference list of the included studies. Finally, 26 studies were included in the final synthesis (Figure 2).

### 3.2 Characteristics of Included Studies

**FIGURE 3:**
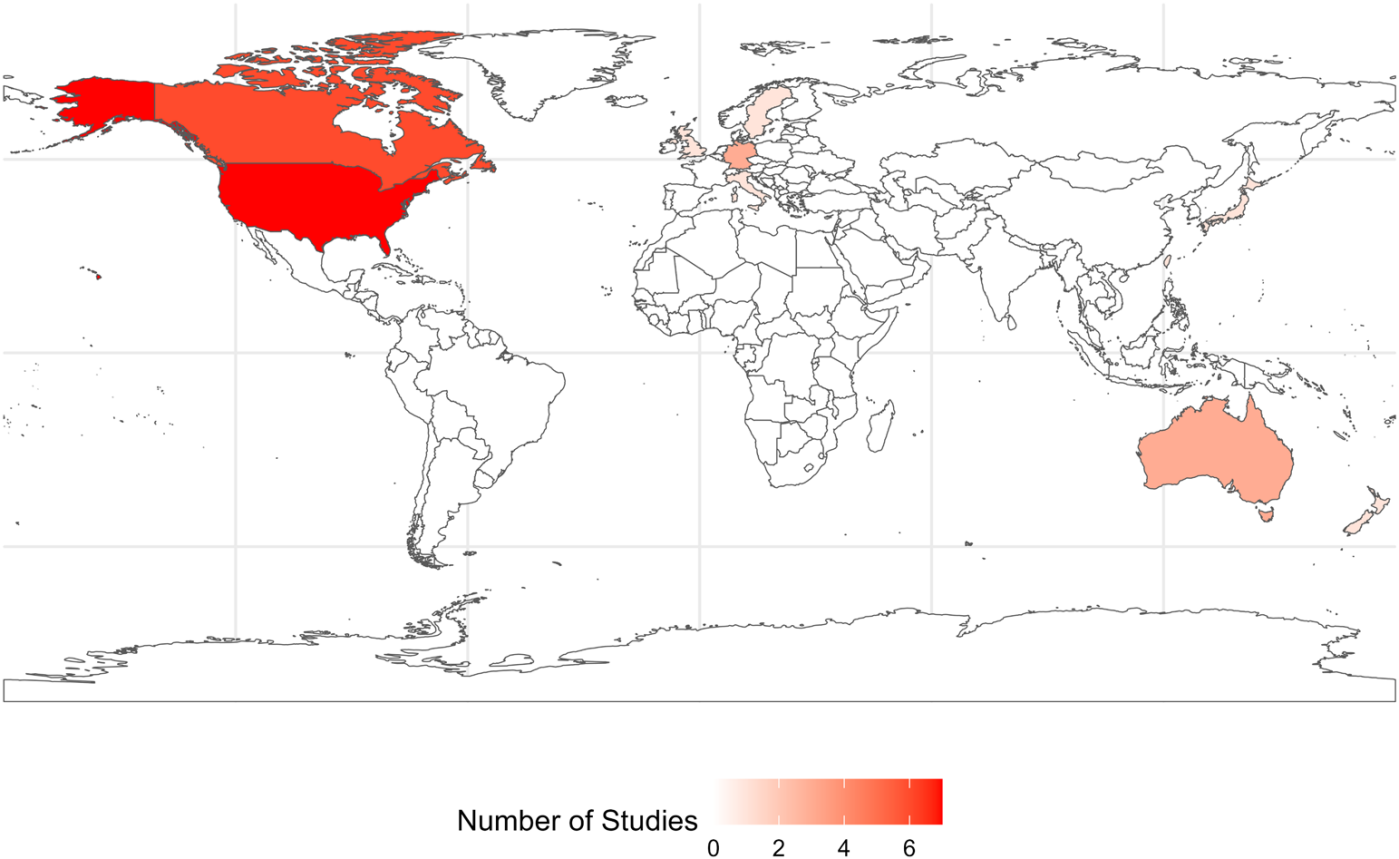
Number of studies on the economic burden associated with eating disorders.

The summary of the main study and methodological characteristics of the 26 included studies are presented in Tables 1 and 2, respectively. Most of the studies were from the United States of America (USA) (seven studies) (Streatfeild et al. 2021; Ballard and Crane 2015; Bellows et al. 2015; Ling, Rascati, and Pawaskar 2017; Patel et al. 2018; Presskreischer, Steinglass, and Anderson 2022; Samnaliev et al. 2015), followed by Canada (six studies) (de Oliveira et al. 2016, 2017, 2023; Gill et al. 2022; Toulany et al. 2015), Germany (three studies) (Bode et al. 2017; Bothe, Walker, and Kroger 2021; Stuhldreher et al. 2015) and Australia (three studies) (Tannous et al. 2021; Gatt et al. 2014). Eligible studies also included one study each from Italy (Marchili et al. 2024), the United Kingdom (UK) (Jenkins 2022), Sweden (Watson et al. 2018), Japan (Kurisu et al. 2023), South Korea (Lee et al. 2021), Taiwan (Tseng, Tu, and Chang 2021), and New Zealand (Surgenor et al. 2022) (Figure 3). Less than one‐third of the studies (seven studies) reported the ethnicity/race of the study population, of which five studies were from the USA (Bellows et al. 2015; Ling, Rascati, and Pawaskar 2017; Patel et al. 2018; Presskreischer, Steinglass, and Anderson 2022; Samnaliev et al. 2015), and one each from the UK (Jenkins 2022) and New Zealand (Surgenor et al. 2022).

**TABLE 1:** Characteristics of included studies.

Author	Country	State/city (if applicable)	Setting	Study period	Study design	Sample size	Study population	Sex	Race/ethnicity	Socioeconomic status	Types of EDs included
Ballard and Crane (2015)	USA		Outpatient	2006–2011	Longitudinal retrospective	5445 with AN (*n* = 1137), BN (*n* = 1869), and EDNOS (*n* = 2439)	Individuals aged 2–71 years	Female: 92.8% Male: 7.1%	NR	NR	AN, BN, and EDNOS
Bellows et al. (2015)	USA	Nationwide	Inpatient and outpatient from the Department of Veterans Affairs (152 medical centers, 1400 clinics, and other centers across the USA)	2005–2011	Retrospective cohort observational	1823	Adults aged 18 years and above	Female: 31.9% Male: 69.1%	White: 70.2% Black: 17% Hispanic: 2.2% Other/unknown: 10.6%	NR	BED, EDNOS without BED
Bode et al. (2017)	Germany	Nationwide	Not clear	2010	N/A	N/A	Not clear	NR	NR	NR	AN and BN
Bothe, Walker, and Kroger (2021)	Germany	Nationwide	Patient‐level claim data from 70 nationwide statutory health insurance providers	2013–15	Retrospective observational	2481 with AN (*n* = 1313) and BN (*n* = 1168)	Patients aged 10–65 years	Female: 94.5% Male: 5.5%	NR	NR	AN and BN
Butterfly Foundation (2024)	Australia	Nationwide	Inpatient, emergency department, outpatient and primary care	2022–23	Literature‐based data	N/A	Individuals aged 5 years and above	Males and females	NR	NR	AN, BN, OSFED, and UFED
Deloitte Access Economics (2023)	Canada	Inpatient hospitalization: nationwide Emergency department: Ontario, Quebec and Alberta Outpatient: Alberta	Inpatient, emergency department, and outpatient	2019–2022	Literature‐based data	N/A	Children and youth aged 5–25 years	Males and female	NR	NR	AN, BN, OSFED, and UFED
de Oliveira et al. (2016)	Canada	Ontario	Inpatient	2003–2011	Retrospective observational	Patient with EDs (*n* = 286) Control (*n* = 1144)	Patient with mean age 24.2 years (age range NR)	Female: ~94.5% Male: ~5.5%	NR	Neighborhood income quintile (low to high) 1st quintile: 12.2% 2nd quintile: 11.6% 3rd quintile: 19.2% 4th quintile: 20.3% 5th quintile: 36.7%	AN, BN, and EDNOS
de Oliveira et al. (2017)	Canada	Ontario	Inpatient	2012	Retrospective observational	Case (*n* = 6326) Control (12,652)	Individuals aged over 4 years old	Female: 92.7% Male: 7.3%	NR	Neighborhood income quintile (low to high)^c^ 1st quintile: 19.3% 2nd quintile: 18.1% 3rd quintile: 17.8% 4th quintile: 21.7% 5th quintile: 22.8% Missing: 0.4%	AN, BN, and EDNOS
de Oliveira et al. (2023)	Canada	Ontario	Inpatient	2019	Cross‐sectional retrospective observational	7547	Individuals aged 18 years or over	Female: 93% Male: 7%	NR	Income quintile (low to high): 0.4%, 19.4%, 19.8%, 17.9%, 19.6%, and 22.9%	AN, BN, and EDNOS
Gatt et al. (2014)	Australia	New South Wales	Outpatient (2 hospital‐based clinics)	2009–12	Prospective observational study	90	Patients with a mean age of 24.5 years	Female: 98.9% Male: 1.1%	NR	Education status (secondary school or lower 21.3%, University or TAFE 78.7%) Employment status (employed 54.5%, unemployed 31.1%, other 14.4%) Private health insurance (Yes 86.7%, No: 13.3%) Income Less than AUD 20,000: 10.1% AUD 20,000‐39,999: 21.3% AUD 40,000‐59,999: 7.9% AUD 60,000‐79,999: 7.9% AUD 80,000‐99,999: 7.9% AUD 100,000 or more: 24.4% Do not know/rather not answer: 20.2%	AN, BN, BED, EDNOS, and type not known
Gill et al. (2022)	Canada	Ontario	Inpatient (165 general and pediatric hospitals)	2014–2019	Cross‐sectional retrospective observational	Not clear	NR for AN	Both female and male, with no percentage reported for AN	NR	NR for AN	AN
Jenkins (2022)	UK	Buckinghamshire, Oxfordshire, and Wiltshire	Three specialist ED services	NR	Retrospective observational	126	Adults with a mean age of 30.2 years	Female: 93.7% Male: 6.3%	White—British: 82.5% White—other: 11.1% Mixed 1.6% Not stated: 1.6%	Employment status (employed 63.5%, unemployed 5.6%, full‐time student 27%, and other 4%)	Nonunderweight BED
Kurisu et al. (2023)	Japan	Tokyo, Osaka, Kyoto, and Chiba	Outpatient (Psychosomatic Medicine department of three centers and the Psychiatry department of another three centers)	2015–17	Prospective observational study	256	Individuals aged 11–55 years	Female: 87.1% Male: 2.7% Missing: 10.2%	NR	NR	AN, BN, BED, and other EDs (DSM‐5)/types not known
Lee et al. (2021)	South Korea	Nationwide	Inpatient and outpatient	2010–15	Retrospective cross‐sectional observational	NR	Individuals with EDs (age group NR)	Female and male (proportion NR)	NR	NR	AN, BN, other EDs and unspecified EDs
Ling, Rascati, and Pawaskar (2017)	USA	Nationwide	Online survey	2013	Retrospective observational	1720	Individuals aged 18 years and older	Female^c^: 29.7%^c^ Male: 70.3%	White^c^: 83.1% Black: 9.3% Other: 7.6%	Income^c^ < USD 25 K: 20.9% USD 25 50 K: 27.9% US 50‐75 K: 23% USD 75 K or more: 25.3%. Decline to answer: 2.9% Education^c^ college degree: 46.8% Less than college degree: 53.2%	BED
Marchili et al. (2024)	Italy	IRCCS Bambino Gesu Children Hospital, Rome	Inpatient	2019–20	Retrospective cross‐sectional observational	260	Children and adolescent aged 6–18 years	Female 93.5% Male 6.5%	NR	NR	AN
Patel et al. (2018)	USA	Nationwide	Inpatient	2010–14	Retrospective observational	3319	Individuals aged 1–80 years	Female: 91.7% to 96.9% in different study Male: 3.1% to 11% in different study years	White: 74.7% to 89.9% Black: 3.2% to 5.1% Hispanic: 2.6% to 15.2% Asian: 1% to 3.3% Native American: 0% to 2%	Health insurance status (private insurance 55.7%, Medicaid 23.8%, other 20.5%)	BN
Presskreischer, Steinglass, and Anderson (2022)	USA	Nationwide	Inpatient, outpatient, carrier, and home health setting (United States Medicare and Medicare Advantage patients)	2016	Cross‐sectional observational	11,962,287, with 17,974 having any type of EDs and 11,944,313 without any EDs	Males and females of all age groups (age range NR)	Female: 73.8% Male: 26.2%	White: 74.6% Black: 10.4% Hispanic: 9% Other/unknown: 5.9%	NR	AN, BN, BED, OSFED, and UFED
Samnaliev et al. (2015)	USA	Nationwide	Survey	2007–11	Retrospective observational panel survey	168,951 individuals, with 86 having EDs	0–85 years men and women	Female: 36.1% Male: 63.9%	White: 82.7% Other: 17.3%	Education mean education: 13 years	AN, BN, BED, Other EDs (according to ICD‐9)
Streatfeild et al. (2021)	USA	Nationwide	Inpatient, emergency department, outpatient, primary care, and residential care	2018–19	Literature‐based data	N/A	Males and females aged 13 years and over	Female and male (proportion NR)	NR	NR	AN, BN, BED, and OSFED (costs of EDNOS reported under this category)
Stuhldreher et al. (2015)	Germany	Dortmund/Bochum, Erlangen, Essen, Freiburg, Hamburg, Heidelberg, Munchen, Munster, Tubingen and Ulm	10 outpatient departments of university departments of psychosomatic medicine and psychotherapy	Not clear	Randomized controlled design	225	Females aged 18 years or older	Female: 100%	NR	NR	AN
Surgenor et al. (2022)	New Zealand	Online survey nationwide	Survey	2016–20	Cross‐sectional survey	121^a^	Affected individuals aged 12–51 years^a^ carers aged 17–73 years	Affected individual: Female^b^ 92.6% Male 7.4% Carers: Female 83.5% Male 7.4% Other/prefer not to say 9.1%^a^	NZ born European 89.3%, other European 3.3%, Maori 3.5%, and Other 7.2%^b^	NR	AN, BN, BED, and OSFED
Tannous et al. (2021)	Australia	South Australia	Household survey	2017	Cross‐sectional	2977	Individuals aged 15 years and above	Female: 58.6% Male: 31.4%	NR	Education (high school or less 38.4%, trade qualification/certificate/diploma 37.5%, and Bachelor of higher 24%) Employment (employed 49.1%, unemployed 8.2%, and not in the labor force 42.7%)	AN, BN, BED, OSFED, and UFED
Toulany et al. (2015)	Canada	Toronto	Inpatient	2011–13	Retrospective cross‐sectional observational	73	Patient aged 12‐18 years	Female: 89% Male: 11%	NR	NR	AN
Tseng, Tu, and Chang (2021)	Taiwan	Nationwide	Inpatient, emergency department, outpatient, and ambulatory care	2002–13	Retrospective observational pooled data	AN (*n* = 1383) and control (*n* = 13,830); BN (*n* = 10,350) and control (103,500)	Individuals aged 18–65 years	AN (female 89.5%, male 10.5%) BN (female 93.6%, male 6.4%)	NR	NR	AN and BN
Watson et al. (2018)	Sweden	Nationwide	Inpatient and outpatient	2005–09	Case–control design	BED (*n* = 319) and control (*n* = 3190)	Individuals aged 14–29 years	Female: 97% Male: 3%	NR	NR	BED

Abbreviations: AN: anorexia nervosa; BED: binge eating disorder; BN: bulimia nervosa; DSM: diagnostic and statistical manual of mental disorders; ED: eating disorder; EDNOS: eating disorders not otherwise specified; NR: not reported; OSFED: other specified feeding and eating disorder; UFED: other unspecified feeding and eating disorder.

^a^ Affected individuals with EDs.

^b^ Carers of affected individuals with EDs.

^c^ Proportions of cases are mentioned that are close to controls.

**TABLE 2:** Methodological characteristics of the included studies.

Author	Type of study	Year of costing	Diagnostic criteria for EDs	Perspective	Epidemiological approach	Direct costing method	Indirect costing method^b^	Discounting (if applicable)^a^	Administrative healthcare utilization data (yes/no)	Self‐reported healthcare utilization (yes/no)
Ballard and Crane (2015)	OCS	Not clear	Claim data	Third‐party (health insurance company)	N/A	Bottom‐up (NR)	N/A	No	Yes	No
Bode et al. (2017)	COI	Not clear	N/A	Not clear	Prevalence‐based	N/A	N/A	N/A	Literature‐based	Literature‐based
Bellows et al. (2015)	OCS	2011	ICD‐9	Not clear	N/A	NR	N/A	No	Yes	No
Bothe, Walker, and Kroger (2021)	OCS	Not clear	ICD‐10	Not clear	N/A	NR	NR	No	Yes	No
Butterfly Foundation (2024)	COI	2022–23	N/A	Societal	Prevalence‐based	Top‐down	Human capital approach	N/A	Yes	No
Deloitte Access Economics (2023)	COI	2023	N/A	Health system	NR	NR	NR	No	Yes	No
de Oliveira et al. (2016)	COI	2012	ICD‐10, ICD‐9, DSM‐4	Public third‐party payer	NR	Bottom‐up	N/A	No	Yes	No
de Oliveira et al. (2017)	COI	2012	ICD‐10, ICD‐9, DSM‐4	Public third‐party payer	Prevalence‐based	Bottom‐up and top‐down	N/A	N/A	Yes	No
de Oliveira et al. (2023)	OCS	2019	ICD‐10, ICD‐9, DSM‐4	Public third‐party payer	Prevalence‐based	NR	N/A	N/A	Yes	No
Gatt et al. (2014)	OCS	Not clear	Self‐reported	Individual/household	NR	NR	N/A	No	No	Yes
Gill et al. (2022)	COI	2018	ICD‐10	Not clear	NR	NR	N/A	No	Yes	No
Jenkins (2022)	COI	2017	Self‐reported	Societal	Prevalence‐based	Bottom‐up	Human capital method	Not clear	No	Yes
Kurisu et al. (2023)	OCS	2017	DSM‐5	Not clear	Prevalence‐based	NR	N/A	No	No	Yes
Lee et al. (2021)	COI	2010–15	ICD‐10	Societal	NR	NR	Human capital method (NR)	No	Yes	No
Ling, Rascati, and Pawaskar (2017)	OCS	2013	DSM‐5	Societal	NR	NR	Human capital method	N/A	No	Yes
Marchili et al. (2024)	OCS	2020–22	DSM‐5	Healthcare system (NR)	NR	NR	N/A	No	Yes	No
Patel et al. (2018)	COI	2010–14	ICD‐9	Not clear	NR	NR	N/A	No	Yes	No
Presskreischer, Steinglass, and Anderson (2022)	OCS	2016	ICD‐10	Not clear	N/A	NR	N/A	N/A	Yes	No
Samnaliev et al. (2015)	OCS	2011	ICD‐9	Not clear	N/A	NR	NR	No	Not clear	Not clear
Streatfeild et al. (2021)	COI	2018–19	N/A	Societal	Prevalence‐based	Bottom‐up	Human capital method	N/A	Literature‐based	Literature‐based
Stuhldreher et al. (2015)	OCS	2008	Eating Disorder Inventory‐2	Societal	NR	NR	Opportunity cost approach for informal care	Not clear	No	Yes
Surgenor et al. (2022)	OCS	2016–20	Self‐reported (definitions were provided using key DSM‐5 criteria)	Societal (NR)	NR	NR	Opportunity cost approach	No	No	Yes
Tannous et al. (2021)	COI	2018	DSM‐5	Societal	Prevalence‐based	Bottom‐up	Human capital method	N/A	No	Yes
Toulany et al. (2015)	OCS	2013	DSM‐4, psychometric tests and comprehensive clinical assessments	Societal	N/A	Bottom‐up	Human capital method	No	Yes	No
Tseng, Tu, and Chang (2021)	OCS	Not clear	ICD‐9	Healthcare system (NR)	N/A	Bottom‐up (NR)	N/A	No	Yes	No
Watson et al. (2018)	OCS	2015	DSM‐4, ICD‐10	Healthcare system (NR)	N/A	Bottom‐up (NR)	N/A	No	Yes	No

Abbreviations: COI: cost of illness; DSM: diagnostic and statistical manual of mental disorders; ICD: international classification of diseases; N/A: not applicable; NR: not reported; OCS: other cost studies.

^a^ Applicable if the study period is more than 1 year.

^b^ The indirect cost using the human capital method estimates the value of human capital as the present value of future earnings, assuming that future earnings serve as a proxy for future productivity. The opportunity cost approach considered the economic value of lost leisure time in providing informal care.

Economic cost evidence was available in 12 studies for AN (Streatfeild et al. 2021; Tannous et al. 2021; Ballard and Crane 2015; Gill et al. 2022; Toulany et al. 2015; Bode et al. 2017; Stuhldreher et al. 2015; Gatt et al. 2014; Marchili et al. 2024; Lee et al. 2021; Tseng, Tu, and Chang 2021; Butterfly Foundation 2024), 11 studies for BN (Streatfeild et al. 2021; Tannous et al. 2021; Ballard and Crane 2015; Patel et al. 2018; Bode et al. 2017; Bothe, Walker, and Kroger 2021; Gatt et al. 2014; Jenkins 2022; Lee et al. 2021; Tseng, Tu, and Chang 2021; Butterfly Foundation 2024), six studies for BED (Streatfeild et al. 2021; Tannous et al. 2021; Bellows et al. 2015; Ling, Rascati, and Pawaskar 2017; Jenkins 2022; Butterfly Foundation 2024), seven studies for other specified eating or feeding disorders (OSFED), unspecified feeding or eating disorders (UFED), or eating disorders not otherwise classified (EDNOS) (Streatfeild et al. 2021; Tannous et al. 2021; Ballard and Crane 2015; Bellows et al. 2015; Jenkins 2022; Lee et al. 2021; Butterfly Foundation 2024), and 12 studies for EDs all together (Streatfeild et al. 2021; Tannous et al. 2021; Presskreischer, Steinglass, and Anderson 2022; Samnaliev et al. 2015; de Oliveira et al. 2016, 2017, 2023; Kurisu et al. 2023; Lee et al. 2021; Surgenor et al. 2022; Butterfly Foundation 2024; Deloitte Access Economics 2023) (Table 1).

More than one‐third of the included studies (10 studies) were conducted from the societal perspective (Streatfeild et al. 2021; Tannous et al. 2021; Ling, Rascati, and Pawaskar 2017; Toulany et al. 2015; Bothe, Walker, and Kroger 2021; Stuhldreher et al. 2015; Jenkins 2022; Lee et al. 2021; Surgenor et al. 2022; Butterfly Foundation 2024), with seven studies being conducted from the health system perspective (de Oliveira et al. 2016, 2017, 2023; Marchili et al. 2024; Watson et al. 2018; Tseng, Tu, and Chang 2021; Deloitte Access Economics 2023). One study adopted third‐party (health insurance) (Ballard and Crane 2015), and another study used the individual payer's perspective (Gatt et al. 2014). The study perspective of the rest of the studies was not clear (seven studies) (Bellows et al. 2015; Patel et al. 2018; Presskreischer, Steinglass, and Anderson 2022; Samnaliev et al. 2015; Gill et al. 2022; Bode et al. 2017; Kurisu et al. 2023).

A prevalence‐based epidemiological method was commonly used (eight studies, 31%) in cost studies contained in this systematic review (Streatfeild et al. 2021; Tannous et al. 2021; de Oliveira et al. 2017, 2023; Bode et al. 2017; Jenkins 2022; Kurisu et al. 2023; Butterfly Foundation 2024). While some studies adjusted for inflation, where applicable (Tannous et al. 2021; Bellows et al. 2015; Samnaliev et al. 2015; de Oliveira et al. 2016; Gill et al. 2022; Watson et al. 2018), most did not apply any discounting of the costs (accounting for differences in cost value at different time points) where the time horizon was more than 1 year. Five studies adopted the “bottom‐up” costing method (a technique that employs comprehensive and precise data on the usage of services and resources at the provider level to determine the cost of individual units (Jo 2014; Tarricone 2006; Chapko et al. 2009)) to estimate direct costs (Streatfeild et al. 2021; Tannous et al. 2021; de Oliveira et al. 2016; Toulany et al. 2015; Jenkins 2022), while one study (de Oliveira et al. 2017) adopted both the “bottom‐up” and the “top‐down” costing method (an approach that relies on overall healthcare expenditures and specific disease rates to illustrate the costs associated with each disease [Jo 2014; Tarricone 2006]). One study adopted only top‐down costing method (Butterfly Foundation 2024). Eighteen studies did not report the direct costing method applied (Ballard and Crane 2015; Bellows et al. 2015; Ling, Rascati, and Pawaskar 2017; Patel et al. 2018; Presskreischer, Steinglass, and Anderson 2022; Samnaliev et al. 2015; de Oliveira et al. 2023; Gill et al. 2022; Bothe, Walker, and Kroger 2021; Stuhldreher et al. 2015; Gatt et al. 2014; Marchili et al. 2024; Watson et al. 2018; Kurisu et al. 2023; Lee et al. 2021; Tseng, Tu, and Chang 2021; Surgenor et al. 2022; Deloitte Access Economics 2023). Studies conducted from the societal perspective adopted the human capital approach (a method that assesses the worth of human capital by determining the present value of an individual's future earnings, with the assumption that these earnings serve as a proxy for future productivity [Jo 2014; Tarricone 2006]) to estimate indirect costs of the studies (Streatfeild et al. 2021; Tannous et al. 2021; Ling, Rascati, and Pawaskar 2017; Toulany et al. 2015; Jenkins 2022; Butterfly Foundation 2024).

Health administrative data were utilized in more than half of the studies (16 studies) (Ballard and Crane 2015; Bellows et al. 2015; Patel et al. 2018; Presskreischer, Steinglass, and Anderson 2022; de Oliveira et al. 2016, 2017, 2023; Gill et al. 2022; Toulany et al. 2015; Bothe, Walker, and Kroger 2021; Marchili et al. 2024; Watson et al. 2018; Lee et al. 2021; Tseng, Tu, and Chang 2021; Butterfly Foundation 2024; Deloitte Access Economics 2023), with another seven studies based on surveys (Tannous et al. 2021; Ling, Rascati, and Pawaskar 2017; Stuhldreher et al. 2015; Gatt et al. 2014; Jenkins 2022; Kurisu et al. 2023; Surgenor et al. 2022) and two study sourced data from the literature (Streatfeild et al. 2021; Bode et al. 2017). The data source were not clear in one study (Samnaliev et al. 2015). Out of 26 studies, 11 studies met the criteria for COI studies (Streatfeild et al. 2021; Tannous et al. 2021; Bellows et al. 2015; Patel et al. 2018; Presskreischer, Steinglass, and Anderson 2022; Samnaliev et al. 2015; de Oliveira et al. 2016, 2017; Gill et al. 2022; Bode et al. 2017; Jenkins 2022; Kurisu et al. 2023; Lee et al. 2021; Butterfly Foundation 2024; Deloitte Access Economics 2023).

### 3.3 Cost Components of the Included Studies

The cost components of the included studies are presented in Table 3. Included cost components varied across studies and are discussed in terms of estimated direct and indirect costs below.

**TABLE 3:** Cost categories^a^ in economic cost studies of eating disorders.

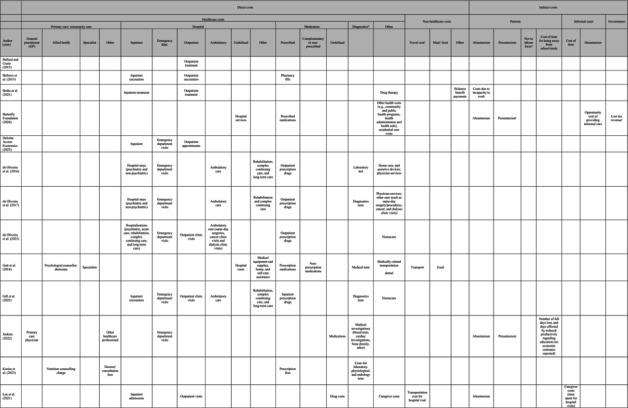
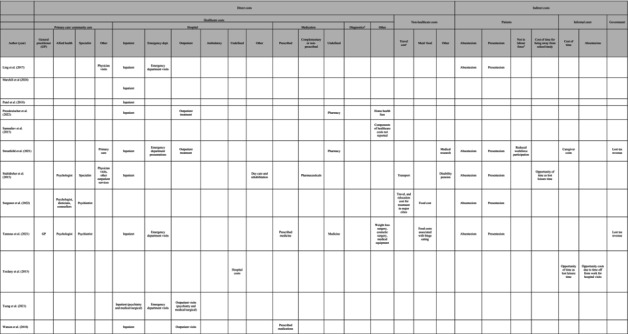

^a^ Cost categories are shown for tangible costs. Bode et al. (2017) only reported intangible costs, and therefore no cost categories for this study are shown.

^b^ Diagnostic can include pathology, radiology and clinical test, screening test, and so on.

^c^ Travel cost associated with receiving medical care.

^d^ Not in labour force due to eating disorders (EDs).

^e^ Income lost due to premature morality because of EDs were also estimated. Other indirect costs included search and hiring costs.

^f^ Other efficiency loss included increase of taxation revenue to other government program associated with EDs (for example, disability support pension, and jobseeker payment).

#### 3.3.1 Direct Costs

All studies of this review reported estimates of direct costs, except the study by Bode et al. (2017). Direct costs were presented in terms of healthcare costs and nonhealthcare costs (but incurred due to receiving care for an ED) in Table 3. Healthcare costs included costs related to primary and community care (general practitioner (GP), allied health, specialist), hospital‐related costs (inpatient encounters, emergency department visits, outpatient visits, rehabilitation, complex continuing care and long‐term care, ambulatory care), costs of medications (prescribed, nonprescribed), diagnostic costs (pathology, radiology, clinical test, screening test), and other costs (costs of weight loss surgery, cosmetic surgery and medical equipment, caregivers' costs etc). Nonhealthcare costs included travel costs associated with receiving healthcare for an ED, meal costs, sickness benefit payments, and disability pension.

#### 3.3.2 Indirect Costs

Nine studies out of 26 studies reported indirect costs associated with EDs in this review (Table 3). Indirect cost components considered were productivity losses due to absenteeism (patients and carers), presenteeism, cost of time for carers, and lost tax revenue. Bothe, Walker, and Kroger (2021) included absenteeism costs due to incapacity to work for individuals aged 18 years and over as the only indirect cost component. However, Lee et al. (2021) included caregiver costs (time spent on hospital visits) in addition to absenteeism costs. Other studies that included carers' opportunity costs due to time off from work for hospital visits (absenteeism) and opportunity of time as lost leisure time was by Toulany et al. (2015) and Butterfly Foundation (2024) report. Six studies reported both presenteeism and absenteeism costs of patients with different types of EDS (Tannous et al. 2021; Ling, Rascati, and Pawaskar 2017; Stuhldreher et al. 2015; Jenkins 2022; Surgenor et al. 2022; Butterfly Foundation 2024). Economic costs of lost tax revenue due to absenteeism and presenteeism were reported in only two studies (Tannous et al. 2021; Butterfly Foundation 2024). Although one study estimated the number of full days lost and days affected by reduced productivity associated with nonunderweight BED impacts on education, no estimates in dollar terms were reported (Jenkins 2022). Only one study estimated the wellbeing loss due to waiting time for seeking ED‐related healthcare during the Covid‐19 period (Deloitte Access Economics 2023).

### 3.4 Cost Estimates

Cost estimates reported in the included studies are discussed in terms of total annual cost for a jurisdiction, total annual costs per‐patient with EDs, and the different types of EDs. These costs are also discussed in relation to tangible costs (direct costs and indirect costs) and intangible costs.

#### 3.4.1 Total Annual Costs

We have identified 11 COI studies related to EDs (Streatfeild et al. 2021; Tannous et al. 2021; Patel et al. 2018; de Oliveira et al. 2016, 2017; Gill et al. 2022; Bode et al. 2017; Jenkins 2022; Lee et al. 2021; Butterfly Foundation 2024; Deloitte Access Economics 2023). Cost estimates of COI studies are reported in Table 4 in PPP‐USD. More than half of the COI studies (six studies) were conducted from the societal perspective (Streatfeild et al. 2021; Tannous et al. 2021; Jenkins 2022; Lee et al. 2021; Butterfly Foundation 2024; Deloitte Access Economics 2023), whereas two studies adopted the health system perspective (de Oliveira et al. 2017, 2016), and the perspective was not reported in three studies (Patel et al. 2018; Gill et al. 2022; Bode et al. 2017). Out of 11 COI studies, six studies reported tangible costs (Patel et al. 2018; de Oliveira et al. 2016, 2017; Gill et al. 2022; Jenkins 2022; Lee et al. 2021; Butterfly Foundation 2024; Deloitte Access Economics 2023) and four studies reported both tangible and intangible costs or the burden of disease (Streatfeild et al. 2021; Tannous et al. 2021; Butterfly Foundation 2024; Deloitte Access Economics 2023), whereas one study only reported intangible costs (Bode et al. 2017).

**TABLE 4:** Annual total costs (in million PPP‐USD^a^) associated with eating disorders.

Type of ED	Author	Country	Health system cost	Direct cost	Indirect cost	Total cost
AN	Butterfly Foundation (2024)	Australia	132.7	132.7	593.0	725.7
	Tannous et al. (2021)	Australia	42.6	44.0	84.1	128.2
	Gill et al. (2022)	Canada	38.6			38.6
	Marchili et al. (2024)	Italy	2.5	2.5		2.5
	Lee et al. (2021)	South Korea	1.3	1.6	0.6	2.2
	Streatfeild et al. (2021)	USA	1162.0^b^	1162.0^b^	10,993.2^b^	12,155.0^b^
BN	Butterfly Foundation (2024)	Australia	177.3	1784.7	1962.0	177.3
	Tannous et al. (2021)	Australia	102.6	166.1	147.8	313.9
	Lee et al. (2021)	South Korea	0.9	1.0	0.4	1.4
	Streatfeild et al. (2021)	USA	904.1^b^	904.1^b^	11,476.6^b^	12,380.8^b^
	Patel et al. (2018)	USA	33.3			33.9
BED	Butterfly Foundation (2024)	Australia	7.5	178.3	3347.4	3525.5
	Tannous et al. (2021)	Australia	60.9	116.2	109.6	225.7
	Jenkins (2022)	UK				5691.9
	Streatfeild et al. (2021)	USA	1306.0^b^	1306.0^b^	19,802.2^b^	21,108.1^b^
Other/unspecified EDs/EDNOS	Butterfly Foundation (2024)	Australia	22.8	22.8	8382.2	8405.0
	Tannous et al. (2021)	Australia	624.0	971.4	1494.2	2465.7
	Lee et al. (2021)	South Korea	1.1	1.3	1.4	2.7
	Streatfeild et al. (2021)	USA	1588.5^b^	1588.5^b^	23,238.7^b^	24,827.3^b^
EDs	Butterfly Foundation (2024)	Australia	170.3	554.0	14107.2	14661.2
	Tannous et al. (2021)	Australia	830.1	1297.9	1835.6	3133.5
	Deloitte Access Economics (2023)	Canada	62.7	62.7		62.7
	de Oliveira et al. (2017)^c^	Canada	69.5			69.5
	de Oliveira et al. (2016)	Canada		3.3		3.3
	Lee et al. (2021)	USA	3.2	3.9	2.4	6.3
	Streatfeild et al. (2021)	USA	4960.5	4960.5	65,510.9	70,471.5

Abbreviations: AN: anorexia nervosa; BED: binge eating disorder; BN: bulimia nervosa; ED: eating disorder; EDNOS: eating disorders not otherwise specified; OSFED: other specified feeding and eating disorder; UFED: unspecified feeding and eating disorder.

^a^ All cost estimates were converted into 2024 USD using gross domestic product (GDP) deflator and relevant countries' purchasing power parity (PPP) exchange rates.

^b^ Excess cost.

^c^ de Oliveira et al. (2017) estimated the excess health system cost of EDs as PPP‐USD 53 million.

Out of 11 COI studies, seven studies reported annual costs associated with EDs and the different types of EDs for the country (Streatfeild et al. 2021; Patel et al. 2018; Bode et al. 2017; Jenkins 2022; Lee et al. 2021; Butterfly Foundation 2024; Deloitte Access Economics 2023). Streatfeild et al. (2021) estimated excess total annual costs associated with EDs in the USA for the fiscal year 2018–19 as PPP‐USD 70.5 billion, with the majority of the costs resulting from the indirect costs (PPP‐USD 65.5 billion, 93%). The study included costs of primary care physicians, other healthcare professionals, inpatient admissions, emergency department visits, medications, and medical investigations as direct costs, as well as costs of absenteeism and presenteeism of the patients. The study also reported that the total annual costs were PPP‐USD 12.2 billion, PPP‐USD 12.4 billion, PPP‐USD 21.1 billion, and PPP‐USD 24.8 billion for AN, BN, BED, and OSFED, respectively. In another study based in the USA, Patel et al. (2018) reported the annual total costs of BN as PPP‐USD 33.9 million using the Nationwide Inpatient Sample for the period 2010–2014. Costs in this study were calculated based on inpatient stay for BN but did not include professional fees and noncovered charges. Five other nationwide COI studies were conducted in South Korea, the UK, Germany, Australia and Canada. In a recent study, Butterfly Foundation (2024) published a report on economic burden of EDs for Australia, which is an update of its previous report (Paxton et al. 2012). Total annual economic cost of EDs in Australia in 2022–23 was estimated as AUD 66.9 billion (PPP‐USD 47.2 billion), with AUD 20.8 billion (PPP‐USD 14.7 billion) as financial/tangible cost and AUD 46.1 billion (PPP‐USD 32.5 billion) as nonfinancial/intangible cost. About 87% of the financial costs (AUD 18.1 billion) resulted from productivity loss. In another recent study, Deloitte Access (2023) estimated the economic cost of EDs among Canadian youths aged 5–25 years, both before and after the COVID‐19 period (Deloitte Access Economics 2023). This study included cost components such as inpatient, emergency department, and outpatient expenses, as well as the loss of well‐being due to the waiting time for receiving treatment. The total health system cost was reported to be CAD 73.3 million (PPP‐USD 62.7 million) in 2021–22, representing an increase of CAD 20.2 million from 2019 to 2020. Additionally, the total costs of well‐being were estimated to be CAD 57.5 million (PPP‐USD 49 million) in 2021–22, reflecting an increase of CAD 19.3 million during the same period. Lee et al. (2021) estimated the economic costs associated with EDs in South Korea using nationally representative data for 2010–15. The annual cost of ED was estimated as USD 5.5 million in 2015 (PPP‐USD 6.3 million), with other and unspecified EDs accounting for most of the costs (PPP‐USD 2.7 million, 43%), followed by AN (PPP‐USD 2.2 million, 35%), and BN (PPP‐USD 1.4 million, 22%). Jenkins (2022) estimated the cost of illness associated with nonunderweight BED based on a clinical trial of 126 participants referred to one of the three specialist ED services in the UK. Costs were calculated for 2017 based on self‐reported healthcare utilization and the amount of time lost from work in terms of absence and reduced productivity. They reported that healthcare use and work impairment cost the UK economy approximately PPP‐USD 5.7 billion per annum. While studies in South Korea and the UK reported direct and indirect cost components, the study by Bode et al. (2017) only reported intangible costs of AN and BN for Germany in 2010.

Other COI studies (two studies) reported annual costs associated with EDs for a state (Tannous et al. 2021) or province (de Oliveira et al. 2016, 2017; Gill et al. 2022). Tannous et al. (2021) estimated the annual total costs of EDs based on a survey via face‐to‐face interviews considering both tangible and intangible costs in South Australia for the year 2018. Total annual tangible economic costs of EDs were estimated as PPP‐USD 3.1 billion, with OSFED and UFED comprising most of the costs (PPP‐USD 2.5 billion, 79%), followed by BN (PPP‐USD 0.31billion, 10%), BED (PPP‐USD 0.23 billion, 7%), and AN (PPP‐USD 0.13 billion, 4%). Indirect costs in this study (productivity loss and tax revenue loss) contributed to most of the total tangible costs associated with EDs (PPP‐USD 1.8 billion, 58%).

The rest of the COI studies (two studies) are based in Ontario, Canada. de Oliveira et al. (2016, 2017) conducted two separate economic analyses among patients receiving specialized inpatient care for an ED and reported healthcare costs from the public third‐payer perspective in Ontario, Canada. In‐province costs for patients who received specialized inpatient care out of the region only were approximately PPP‐USD 3.3 million (de Oliveira et al. 2016), whereas annual direct costs of EDs were estimated as USD PPP‐69.5 million per annum for patients ever hospitalized for an ED in Ontario (de Oliveira et al. 2017). In another study, the annual costs of pediatric hospitalization of AN were estimated as USD PPP‐38.6 million in Ontario (Gill et al. 2022).

**FIGURE 4:**
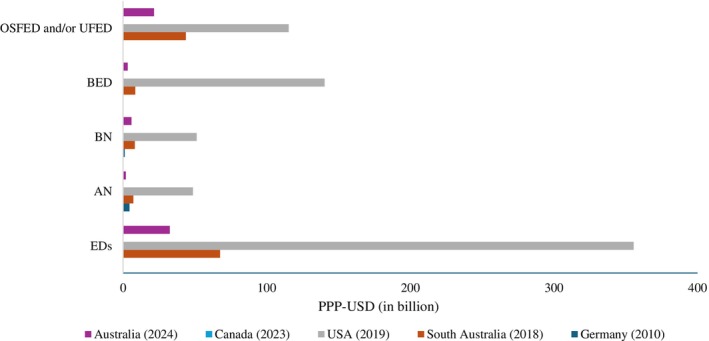
Intangible costs associated with different types of eating disorders (in billion PPP‐USD).

Five studies reported intangible costs (burden of disease) associated with EDs (Streatfeild et al. 2021; Tannous et al. 2021; Bode et al. 2017; Butterfly Foundation 2024; Deloitte Access Economics 2023). In one of these studies, the Butterfly Foundation (2024) estimated the disease burden of EDs for Australia and its types by multiplying disability‐adjusted life years with the value of statistical life years. Both loss of health and premature death due to EDs were incorporated into the calculation. The annual burden of disease of EDs in 2022–23 was estimated as AUD 46.1 billion (PPP‐USD 32.5 billion). Tannous et al. (2021) estimated the disease burden of EDs for the state of South Australia by adopting a similar methodology as the Butterfly Foundation (2024) report based on self‐reported data. The disease burden was estimated as PPP‐USD 67.5 billion for EDs, PPP‐USD 7.1 billion for AN, PPP‐USD 8.2 billion for BN, PPP‐USD 8.5 billion for BED, and PPP‐USD 43.7 billion for OSFED and UFED (Figure 4). Over three‐quarters of the total disease burden was attributed to years lived with disability (YLDs). Using a similar approach as Tannous et al. (2021), Streatfeild et al. (2021) estimated the intangible costs of EDs for the USA, with about 70% of the burden attributed to YLDs in 2018–19. The annual burden of disease due to EDs was estimated as PPP‐USD 355.6 billion. Most of this burden was attributed to BED (PPP‐USD 140.3 billion), followed by OSFED (PPP‐USD 115.3 billion), BN (PPP‐USD 51.3 billion), and AN (PPP‐USD 48.7 billion) (Figure 4). However, Bode et al. (2017) used quality‐adjusted life years to calculate the disease burden of untreated AN and BN in Germany in 2010. Their study estimated the cost of untreated AN and BN as PPP‐USD 4.5 billion and PPP‐USD 1.2 billion, respectively (Figure 4). The only study that estimated total costs of well‐being due to waiting time of receiving treatment was the study based in Canadia that involved patients aged 5–25 years (Deloitte Access Economics 2023). The cost was estimated to be CAD 57.5 million (PPP‐USD 49 million) in 2021–22.

#### 3.4.2 Total Annual Costs Per‐Patient

**FIGURE 5:**
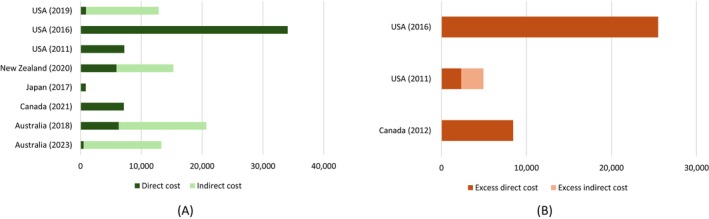
Annual cost/excess cost per‐patient associated with eating disorders (in PPP‐USD).

Eight studies reported annual costs per‐patient for EDs. Among these studies, three of the studies estimated the incremental/excess costs due to EDs. Annual per‐patient costs/excess costs associated with EDs are shown in Figure 5 (costs are presented in Table S6). The annual per‐patient cost associated with EDs varied from PPP‐USD 12,858 to PPP‐USD 41,811 in the USA. In Canada, the annual per‐patient cost of EDs was PPP‐USD 7124, whereas the reported per‐patient cost in Japan was PPP‐USD 862. In Australia, the per‐patient cost associated with EDs was estimated to be up to PPP‐USD 20,698. Indirect cost was the major component (70%–93%) of the total costs in all studies that were conducted from the societal perspective.

Out of 26 studies, excess cost per‐patient was reported in three studies, two of which were conducted in the USA, and the other was conducted in Canada. In the USA, the excess cost per‐patient with EDs was estimated to be up to PPP‐USD 25,491, while the excess cost per‐patient was PPP‐USD 8434 in Canada.

**FIGURE 6:**
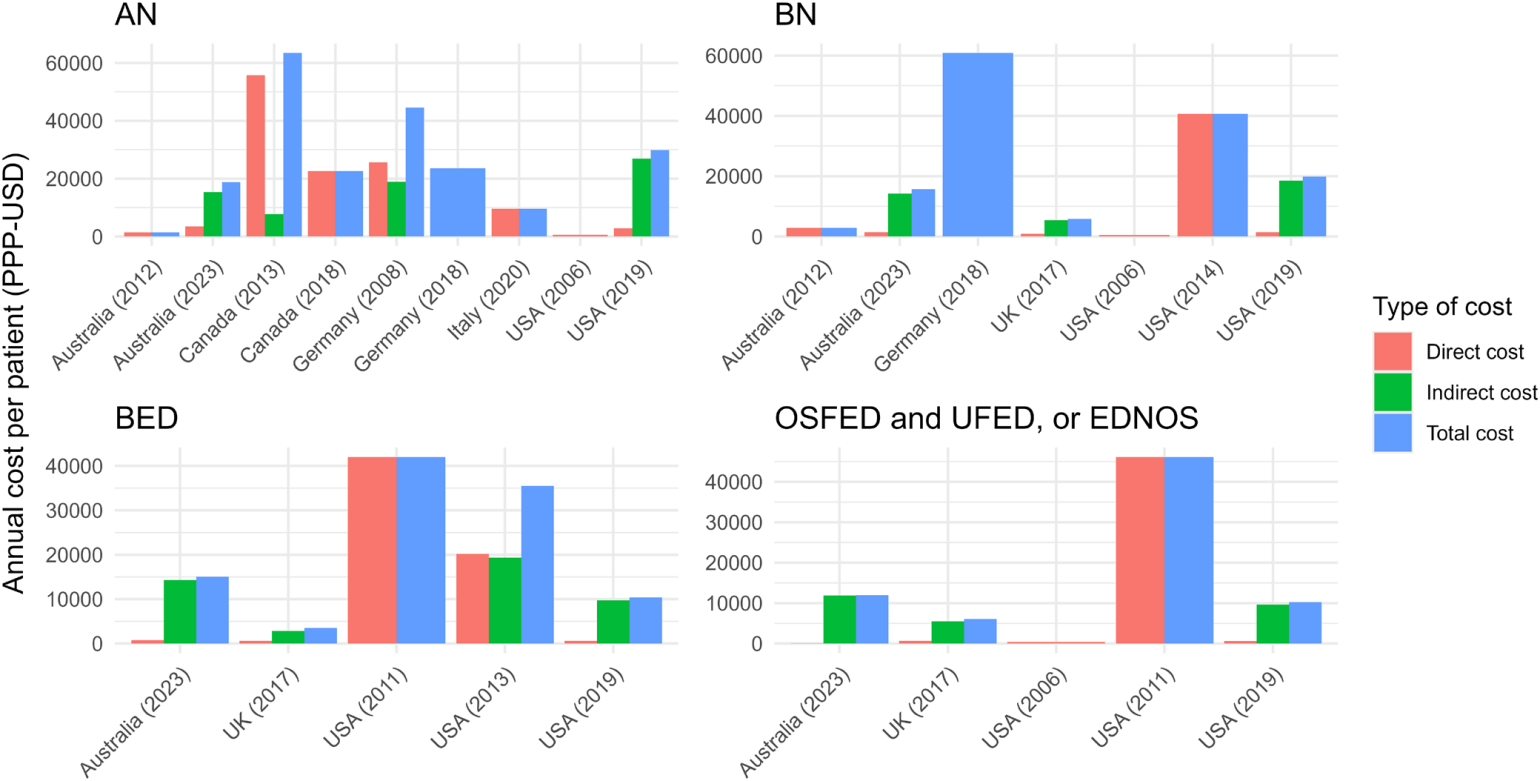
Annual cost per‐patient associated with different types of eating disorders (in PPP‐USD).

In Figure 6, a comparison of annual per‐patient costs associated with different types of EDs is shown, and cost estimate figures are presented in Tables S7 and S8. AN was found to have the highest annual per‐patient cost in Canada, totalling PPP‐USD 63,491 annually per‐patient. In most studies, annual per‐patient costs for Canada, Germany, Australia and the USA were no less than PPP‐USD 18,000. The per‐patient cost for BN varied from PPP‐USD 2862 to PPP‐USD 40,588 across Australia, Germany, the UK, and the USA. Annual per‐patient costs associated with BED were reported in five studies, of which three studies were from the USA. While the annual per‐patient cost for BED in the UK was estimated as PPP‐USD 3517, costs varied from PPP‐USD 10,380 to USD 42,008 in the USA. Costs per‐patient also varied from PPP‐USD 6072 to PPP‐USD 46,165 for other/unspecified EDs across the USA and the UK.

### 3.5 Quality of the Included Studies

The results of the quality assessment of the COI studies are presented in Table 5. Ten out of 11 COI studies were assessed. One study could not be assessed using Schnitzler's checklist as the study only reported intangible costs (Bode et al. 2017). Nearly half (five studies) of the assessed COI studies met the criteria of Schnitzler et al.'s (2023) consensus‐based checklist, either completely or partly, with more than 60% of the checklist elements being fulfilled (Streatfeild et al. 2021; Tannous et al. 2021; de Oliveira et al. 2017; Jenkins 2022; Butterfly Foundation 2022). Study objective, population, perspective, data collection approach, identification and measurement of cost components, valuation in monetary terms, time horizon, cost sectors, and limitations of the study, were among the checklist items, which have been satisfied by at least 70% of the studies. About half of the COI studies did not report on epidemiological and costing approaches.

**TABLE 5:** Quality Assessment of cost‐of‐illness studies^a^ using the Schnitzler et al. (2023) checklist.

		Butterfly Foundation (2024)	Deloitte Access (2023)	de Oliveira et al. (2016)	de Oliveira et al. (2017)	Gill et al. (2022)	Jenkins (2022)	Lee et al. (2021)	Patel et al. (2018)	Tannous et al. (2021)	Streatfeild et al. (2021)	Percentage of “yes”/“partially yes”
Study characteristics	Question											
Question/objective	1. Is a well‐defined research question or objective stated?	Yes	Yes	Yes	Yes	Yes	Yes	Yes	No	Yes	Yes	90%
Population	2. Is the study population described?	Yes	Yes	Yes	Yes	No	Yes	Partially	Yes	Yes	Unclear	80%
Perspective	3. (a) Is (are) the chosen study perspective(s) stated?	Yes	No	Yes	Yes	No	Yes	Yes	No	Yes	Yes	70%
	(b) If so, is (are) the chosen study perspective(s) justified?	Yes	N/A	Unclear	Unclear	N/A	Yes	Yes	N/A	Yes	Yes	50%
Methodology and cost analysis												
Epidemiological approach	4. Is the epidemiological approach reported (e.g., prevalence, incidence)?	Yes	Unclear	N/A	Yes	Yes	Yes	N/A	No	Yes	Unclear	50%
Costing approach	5. Is the costing approach reported (e.g., top‐down, bottom‐up)?	Yes	No	Yes	Yes	No	Yes	Partially	No	Unclear	Yes	60%
Data collection approach	6. Is the data collection process reported (e.g., prospective, retrospective)?	Yes	Partially	Yes	Yes	No	Yes	Yes	Yes	Yes	N/A	80%
Identification	7. (a) Are all components of resource use identified that are relevant to the condition/disease, population, intervention, study objectives, and study perspective?	Yes	Partially	Partially	Partially	Unclear	Partially	Partially	Unclear	Partially	Partially	80%
	(b) If not, is a justification provided for excluding relevant components of resource use?	Yes	No	No	No	No	Yes	No	No	Yes	Yes	40%
Measurement	8. (a) Are all included components of resource use measured?	Yes	Unclear	Yes	Yes	Unclear	Yes	Yes	Unclear	Yes	Yes	70%
	(b) If not, is a justification provided for not measuring certain components of resource use?	N/A	N/A	N/A	N/A	N/A	N/A	N/A	N/A	N/A	N/A	0%
Valuation	9. (a) Are all included components of resource use valued in monetary terms?	Yes	Unclear	Partially	Partially	Partially	Partially	Partially	Unclear	Yes	Yes	80%
	(b) If not, is a justification provided for not valuing certain components of resource use?	N/A	N/A	No	No	No	Yes	No	No	N/A	N/A	10%
Time horizon	10. (a) Is the chosen time horizon specified?	Yes	Yes	Yes	Yes	Yes	Yes	Yes	Yes	Yes	Yes	100%
	(b) If so, is the chosen time horizon justified?	Yes	Yes	Yes	Yes	No	No	No	No	No	No	40%
Discounting	11. (a) Are future costs discounted?	N/A	No	No	N/A	No	N/A	No	No	N/A	N/A	0%
	(b) If so, is a justification provided for the discount rate?	Not appliable	N/A	N/A	N/A	N/A	N/A	N/A	N/A	N/A	N/A	0%
Sensitivity	12. (a) Are all variables whose values are uncertain subjected to sensitivity analysis?	No	No	Yes	Yes	No	Yes	Yes	No	Partially	Yes	60%
	(b) If so, is a justification provided for which variables are subjected to sensitivity analysis?	N/A	N/A	Yes	Yes	N/A	Yes	Yes	No	Yes	Yes	60%
	(c) Are analyses done on relevant subgroups?	Yes	No	Yes	Yes	No	No	No	No	Yes	Yes	50%
Results and reporting												
Cost sectors	13. Are the study results presented transparently by cost category/sector?	Yes	Yes	Partially	Partially	Unclear	Yes	Yes	No	Yes	Yes	80%
Generalizability	14. Do the authors discuss the generalizability of study results (e.g., comparing the results to other patient/client groups or/in other settings)?	Yes	Yes	No	No	No	Partially	No	No	Yes	Yes	50%
Limitations	15. Do the authors discuss important limitations?	Yes	Yes	Yes	Yes	Yes	Yes	Yes		Yes	Yes	90%
Ethical and distributional issues	16. (a) Do the authors discuss ethical issues?	No	No	No	No	No	No	Unclear	No	No	No	0%
	(b) Do the authors discuss distributional issues?	No	No	No	No	No	No	No	No	No	No	0%
Conflict of interest	17. Do the authors report any potential conflicts of interest?	No	No	No	No	Yes	Yes	Yes	Yes	Yes	Yes	60%
Percentage of “yes”/“partially yes”		65%	35%	58%	62%	23%	73%	58%	15%	69%	62%	

Abbreviation: N/A: not applicable.

^a^ The study by Bode et al. (2017) was not assessed as it only reported the monetary value of intangible costs.

## 4 Discussion

This systematic review aimed to summarize the latest evidence on the economic burden of EDs across the globe and critically appraise the economic methodologies of the included studies. Economic research of this nature is vital for guiding healthcare administrators and policymakers in allocating resources effectively to tackle serious mental and physical health issues. These health conditions not only have detrimental effects on individuals with EDs but also on their families/carers in addition to health and other systems (such as education sector) and thus consequences for society and the economy. By quantifying the economic burden of conditions such as EDs, we provide crucial data that can inform policymakers' decisions to allocate resources more efficiently. This can help to alleviate the likely disproportionate cost burdens placed on individuals, families/carers, employers, and communities. It is crucial to understand the full economic impact of these health issues in order to develop effective strategies to reduce their prevalence and severity, ultimately improving public health outcomes and economic stability. At the same time, the credibility of these studies is important which is discussed in terms of methodological aspects, comparison of findings with previous literature, strengths, and limitations after a summary of the findings is provided. Additionally, the scope for future improvement is addressed.

### 4.1 Summary of the Findings

In our systematic review, 26 studies eligible studies published between August 1, 2013, and June 30, 2024, were assessed. Economic cost evidence was available in 12 studies for AN, 11 studies for BN, six studies for BED, seven studies for OSFED, UFED, or EDNOS; and 12 studies for EDs altogether. Out of 26 studies, 11 were COI studies, whereas 15 were classified as OCS. The national total annual tangible cost of EDs was found to be up to PPP‐USD 70.5 billion, with AN having the most per‐patient costs (PPP‐USD 63,491) among all types of EDs. Indirect costs, such as productivity loss, were the major component (70%–93%) of the total costs in all studies that were conducted from the societal perspective. Intangible costs (burden of disease costs) were estimated to be up to PPP‐USD 355.6 billion, with about three‐fourths of the burden attributed to YLDs. Less than half (five studies) of the assessed COI studies met the criteria of Schnitzler et al. (2023) consensus‐based checklist, either completely or partly, with more than 60% of the checklist elements being fulfilled.

### 4.2 Comparison of Findings With Previous Systematic Reviews

From previous systematic reviews (search performed until July 2013) or literature reviews (Simon, Schmidt, and Pilling 2005; Agh et al. 2015, 2016; Stuhldreher et al. 2012), six COI studies (Dickerson et al. 2011; Grenon et al. 2010; Krauth, Buser, and Vogel 2002; Mathers, Vos, and Stevenson 2001; Rathner and Rainer 1997; Nielsen, Moller‐Madsen, and Nystrup 1996) were identified that reported nationwide/regional total annual costs associated with EDs or specific types of EDs. In addition, our systematic reviews identified 11 COI studies related to EDs, which shows that the number of COI studies more than doubled in the last 10 years (August 2013–June 2024) compared with previous years (1980‐July 2023). However, some COI analysis studies could have been omitted due to the exclusion criteria of the previous systematic reviews. For example, the (Paxton et al. 2012) estimated both tangible and intangible costs associated with EDs in Australia, which was not included in the previous systematic reviews (Paxton et al. 2012). Even with this omission, there is notable growth in the number of studies across different types of EDs. Before the study period of this systematic review, COI studies were limited to the UK, Germany, Austria, Australia, and Denmark. The number of COI studies increased for Canada (none to three studies), the USA (none to two studies), and South Korea (none to one study), while studies updated estimates on economic costs for Australia (three new studies) and the UK (one new study). No studies have been found in developing countries. Five studies that reported intangible costs associated with EDs were also published during the last 10 years for Australia (Tannous et al. 2021; Butterfly Foundation 2024), Canada (Deloitte Access Economics 2023), Germany (Bode et al. 2017) and the USA (Stuhldreher et al. 2012), compared with two study published between 1980 and 2013 for Australia (Paxton et al. 2012; Mathers, Vos, and Stevenson 2001).

Overall, studies included in this systematic review lacked reporting on ethnicity or other aspects of diversity, and even the few studies that presented the distribution population by race/ethnicity, none of them reported the costs of EDs by different ethnicity/race sub‐groups. Of the studies that reported the distribution by different race/ethnicity measures, most were from the USA (five out of seven studies). Previous systematic reviews did not report on the race/ethnicity information of the populations considered in the included studies, and no cost analysis for these types of sub‐groups is available in studies in previous systematic reviews (Simon, Schmidt, and Pilling 2005; Agh et al. 2015; Ágh et al. 2016; Stuhldreher et al. 2012; van Hoeken and Hoek 2020). One of the reasons for this is the lack of data on ethnicity in the studies. For example, it was highlighted by Renzaho (2023) that Australia lacks a collection of ethnicity data, which may be one of the reasons that there is not enough cost evidence for EDs by different ethnicities. However, it is also evident that there is a lack of evidence on costs of EDs by different ethnicities where data were available.

### 4.3 Methodological Aspects

#### 4.3.1 Methodological Quality

The methodological quality of the reviewed studies was heterogeneous. While about 75% of the COI studies met the criteria of reporting study objective, population, perspective, data collection approach, identification of cost components, valuation in monetary terms, time horizon, cost sectors, study limitations, and conflict of interest, several methodological aspects were not frequently met. About half of the COI studies did not report the epidemiological and economic costing approaches used. Although some studies adjusted for inflation, no study that estimated costs for more than a year applied discounting. Similar to the findings of the previous review, the robustness of the results of the reviewed studies was rarely checked with sensitivity and uncertainty analyses (Stuhldreher et al. 2012).

#### 4.3.2 Identification of Excess Costs

This systematic review revealed a limited number of studies that estimated excess costs due to EDs. This aligns with the previous systematic review, where three studies reported excess costs (Stuhldreher et al. 2012). While the usual costs associated with EDs are important information, they may not show the causal impact of the disease. Understanding excess costs can help healthcare policymakers and administrators make informed decisions about prioritizing health programs and policies that are not only effective but can also reduce overall costs associated with EDs. Excess costs attributed to EDs were estimated, either employing a comparison group (Ling, Rascati, and Pawaskar 2017; Presskreischer, Steinglass, and Anderson 2022; Samnaliev et al. 2015; de Oliveira et al. 2017; Tseng, Tu, and Chang 2021) or using estimates from previous literature (Streatfeild et al. 2021). The lack of literature on excess costs of EDs may be due to the type of data utilized where more rigorous quasi‐experimental design, such as difference‐in‐difference and instrumental variables approach, could not be applied to estimate excess cost/causal economic impact of EDs. This methodological limitation was evident in the reviewed studies of the previous systematic review (Stuhldreher et al. 2012).

#### 4.3.3 Indirect Cost and Intangible Cost Components

The societal costs of EDs are significant. Although these costs have received less attention historically, studies focused on quantifying these are now being conducted more (Crow 2014). Compared to previous systematic reviews (Simon, Schmidt, and Pilling 2005; Stuhldreher et al. 2012), the number of COI studies incorporating indirect costs has increased from one study (Krauth, Buser, and Vogel 2002) to five studies (Tannous et al. 2021; Patel et al. 2018; Jenkins 2022; Lee et al. 2021; Krauth, Buser, and Vogel 2002). This systematic review found that indirect cost was a major component of the estimated total costs, comprising between 25% and 93% of the tangible costs across various studies. Some important indirect cost components, such as the economic value of the number of school days missed and the lifetime economic impact of reduced productivity in education due to EDs, are yet to be estimated. The only study that reported on the number of full days lost and days affected by reduced productivity regarding this gap in education did not progress in estimating the monetary value of this cost (Jenkins 2022). This is an important factor in estimating the costs of EDs as the last few years of childhood and early adolescence is a critical period where the risk factors for EDs become pronounced (Rohde, Stice, and Marti 2015; Smink, Hoeken, and Hoek 2012; Alonso et al. 2005).

While estimates of the global burden of disease for AN, BN, BED, OSFED, and UFED are included in the Global Burden of Diseases, Injuries, and Risk Factors Studies (Santomauro et al. 2021; Whiteford et al. 2013), this intangible component was costed in only a limited number of studies, with only two studies of this systematic review providing a nationwide estimate of this cost for EDs. Up to the conduct of this review, there have only been four nationwide studies that estimated the economic cost of the disease burden of EDs, with one study being conducted for the USA (Streatfeild et al. 2021), one for Germany (Bode et al. 2017) and two for Australia (Paxton et al. 2012; Mathers, Vos, and Stevenson 2001), and two of which are more than a decade old (Paxton et al. 2012; Mathers, Vos, and Stevenson 2001).

#### 4.3.4 Variation in Costs

Considerable variations are evident in the cost estimates across and within countries. For example, in the USA, the total annual costs of BN were found to be PPP‐USD 12.4 billion in the study by Streatfeild et al. (2021) and PPP‐USD 33.3 million in the study by Patel et al. (2018). This difference in the estimates was mainly attributed to the perspective of the study, methodology, cost components and year of the study. While Streatfeild et al. (2021) included costs of inpatient admissions, emergency department presentations, primary and outpatient visits, pharmaceuticals, medical research, productivity costs, and caregiver costs in their calculation, Patel et al. (2018) estimated the inpatient costs only. Variations in annual per‐patient costs were also evident (Patel et al. 2018). Within the USA, the annual per‐patient direct cost associated with EDs varied from PPP‐USD 905 to PPP‐USD 34,071 (Streatfeild et al. 2021; Presskreischer, Steinglass, and Anderson 2022; Samnaliev et al. 2015). The difference in per‐patient direct costs reported by Presskreischer, Steinglass, and Anderson (2022) and Streatfeild et al. (2021) are mainly attributed to differences in the methodology used, as these studies included similar cost components. Importantly, the study by Presskreischer, Steinglass, and Anderson (2022) utilized administrative health data, whereas the study by Streatfeild et al. (2021) used literature‐based data of prevalence and costs. As health administrative data are considered more representative of patients and more accurate (Kim et al. 2023; Kurdyak et al. 2020), the per‐patient cost of EDs is more likely to be estimated accurately in the Presskreischer, Steinglass, and Anderson (2022) study. Even in the countries where most of the healthcare is financed publicly, the cost difference remained notable (PPP‐USD 43,637 in Australia vs. PPP‐USD 10,258 in Canada (Tannous et al. 2021; de Oliveira et al. 2023)). Differences in the methodology, sample population, year of study, and inclusion of different cost components may be the reason for this difference.

### 4.4 Strengths and Limitations

The present study incorporated a systematic search and a comprehensive selection criterion to reduce the potential bias in study selection. The search conducted involved major databases, including major economic and medical databases, and there was no restriction on published language, which are strengths of this systematic review. Additionally, the current systematic review has compared the estimates of total costs and sub‐groups of costs using the PPP exchange rate, which enabled comparisons across and within countries. Moreover, the broad consideration of economic study types, allowed for a detailed and comprehensive capture of ED treatments across health settings, models of care, and policies.

Despite its strengths, the current study has several notable limitations. First, the heterogeneity of the sample population, contexts, and cost components limited the scope for greater comparison. It was not possible to perform a meta‐analysis due to this heterogeneity and lack of studies published in the same country or countries with similar health systems. Second, a limitation of this systematic review is the reliance on translated abstracts, which may have some translation inaccuracies. However, expert health economists carefully considered relevant key terms and employed an inclusive approach in screening the abstracts to avoid bias in the selection of eligible studies.

### 4.5 Implications for Future Research

Several aspects identified in this systematic review could be the focus of future research, especially in terms of reporting cost estimates and the methodological quality of studies. More emphasis should be placed on estimating costs by including indirect and intangible costs to enrich the evidence base. Future studies should update economic estimates associated with EDs and for the different types of EDs to better understand their relative importance among different mental health diseases and other diseases. It is important to note that previous estimates that only accounted for inflation may not accurately reflect the situation in the reporting year. For instance, the economic burden of EDs in Australia has been reported in existing literature as AUD 80.1 billion in 2022 (Butterfly Foundation 2022), which is an inflated figure of 2012 (AUD 69.7 billion in 2012) (Paxton et al. 2012). However, this (inflated) estimate needs to be used with some caution as it only really reflects the healthcare utilization costs of the reporting year (2012). A very recent report suggests that the total economic cost associated with EDs is AUD 66.9 billion in 2023 for Australia, which is a 36% increase from 2012 when compared to a “like‐for‐like” 2012 cost estimate of AUD 49.2 billion (Butterfly Foundation 2024). Future studies in other countries should consider updating cost estimates based on current data.

Estimates of the financial burden associated with binge eating, weight loss efforts, and exercise are notably scarce in the extant literature. Tannous et al. (2021) represent a singular study that encompasses costs related to binge eating and weight loss surgery. This study estimated the costs of binge eating to be AUD 560 million for South Australia in 2018, accounting for more than half of the health system's total expenses, which amounted to AUD 1 billion. Given the substantial proportion of these costs within the overall economic burden of EDs, future research should prioritize the inclusion of these cost components to attain a more precise estimate understanding of the total cost burden associated with binge eating and EDs more generally.

There is scope for improvement in the methodological quality to identify causal impact/excess costs due to EDs and specific types of EDs. The use of linked healthcare administrative data with proper quasi‐experimental design may overcome the issue of biased cost estimation. A guideline on estimating costs for EDs may be developed as some of the costs, such as the economic value of missed school days, are quite specific to this type of disease, with no costing guidance available. The use of a standardized approach/guidance tool to maintain the methodological or reporting quality of future COI studies of EDs is recommended. Implementing a standardized approach for collecting and reporting cost information can improve the comparability and reliability of cost estimates across studies. This includes the use of uniform cost categories, timeframes, and valuation methods. Moreover, greater investment in gathering consensus from policymakers, clinicians, consumers, individuals with lived experience, health economists, and epidemiologists is crucial (and at a global level). Engaging these stakeholders ensures that the cost estimates reflect real‐world scenarios and are relevant to various perspectives and needs. Incorporating productivity costs using tools such as the Work Productivity and Activity Impairment (WPAI) tool (Reilly, Zbrozek, and Dukes 1993) can provide valuable insights into the indirect economic impacts of EDs on individuals and society. Additionally, a focus on evaluating the cost incurred by cohorts based on race/ethnicity (and SES) is lacking, and addressing these gaps in future research may help to better understand the full range of cost drivers and equity matters. By integrating these recommendations, future research can significantly improve the quality and comprehensiveness of cost estimates related to EDs.

## 5 Conclusion

Although there is agreement in the literature that EDs impose a substantial economic burden on healthcare systems, society, and individuals, a finding that was borne out in this systematic review, there are variations in the methods (thus quality) and perspectives used to assess this burden. It is crucial for healthcare administrators/policymakers to understand the magnitude of this burden when setting healthcare priorities and allocating resources to maximize social welfare. However, comprehensive economic burden studies are lacking in many countries or are not updated with recent data. Therefore, it is essential to improve the methodological quality of future research in terms of design, analysis, and reporting of results.

## Author Contributions

**Moin Ahmed:** conceptualization, data curation, formal analysis, investigation, methodology, software, writing – original draft. **Md Deen Islam:** investigation, methodology, validation. **Phillip Aouad:** writing – review and editing. **Jane Miskovic‐Wheatley:** project administration, writing – review and editing. **Stephen Touyz:** funding acquisition, writing – review and editing. **Sarah Maguire:** funding acquisition, writing – review and editing. **Michelle Cunich:** conceptualization, funding acquisition, methodology, supervision, writing – review and editing.

## Conflicts of Interest

The authors declare no conflicts of interest.

## Supporting information


Data S1.

## Data Availability

The data that supports the findings of this study are available in the manuscript and Supporting Information of this article. Data on gross domestic product (GDP) deflator and relevant countries' purchasing power parity (PPP) exchange rates can be found in the World Economic Outlook (WEO) database 2023.
