# Supplementary material for: Global and Regional Economic Burden of Eating Disorders: A Systematic Review and Critique of Methods
Source: Int J Eat Disord. 2024 Nov 14;58(1):91–116. doi: 10.1002/eat.24302 (PMC11784850; doi:10.1002/eat.24302)
Supplement: Supplementary file 1 — Data S1. [file EAT-58-91-s001.docx]

# Supplementary Tables

## Supplementary Table 1: Search strategy in MEDLINE(Ovid)

| 1 | exp "feeding and eating disorders"/ or exp Anorexia Nervosa/ or exp Bulimia Nervosa/ or Anorexia/ or Bulimia/ |
| --- | --- |
| 2 | ("Abnormal Eat*" or Anorexia* or "Anorexia Nervosa" or "Avoidant Restrictive Food" or Binge* or "Bulimia Nervosa" or Bulimia* or "Eating Disorder*" or Rumination or "Restrict* Eat*" or "Atypical Purging" or "Disord* Eat*" or "Eating Path*" or "Night Eating*" or "Patholog* Eat*" or Pica or "Purging Disorder*").ti,ab. |
| 3 | 1 or 2 |
| 4 | costs and cost analysis/ or "cost allocation"/ or "cost of illness"/ or health care costs/ or health expenditures/ |
| 5 | caregiver burden/ or financial stress/ |
| 6 | ("Economic burden" or "Cost of illness" or "Cost of disease" or "Cost of sickness" or "Illness cost*" or Economic cost* or "Burden of illness" or "Burden of sickness" or "Financial burden" or "Medical cost*" or "Indirect cost*" or "Direct cost*").ti,ab. |
| 7 | ("economic study" or "econ* assess*" or "econ* conseq*" or "resource* use*" or "health* resource*" or "resource* utili*" or "health expendit*" or "out of pocket" or out-of-pocket or co-pay* or "time cost*" or "cost of caregiver*" or "Caregiver* cost" or "Societal cost*" or "Productivity cost*" or "Productivity loss*" or "Human capital cost*" or "Caregiv* burden" or "Carer* burden" or "Family burden").ti,ab. |
| 8 | (cost* adj3 (health* or ill* or disease* or time or society)).ti,ab. |
| 9 | (cost* adj2 (analys* or assess*OR study)).ti,ab. |
| 10 | ("Financial Stress" or "cost allocation" or health care cost*).ti,ab. |
| 11 | 4 or 5 or 6 or 7 or 8 or 9 or 10 |
| 12 | 3 and 11 |
| 13 | limit 12 to dt=20130701-20240630 [July 1st, 2013 to June 30th, 2024] |
| 14 | limit 12 to rd=20130701-20240630 [July 1st, 2013 to June 30th, 2024] |
| 15 | 13 or 14 |

Note: Search conducted on July 1, 2024

## Supplementary Table 2: Search strategy in Embase (Ovid)

| 1 | exp eating disorder/ or exp anorexia/ |
| --- | --- |
| 2 | ("Abnormal Eat*" or Anorexia* or "Anorexia Nervosa" or "Avoidant Restrictive Food" or Binge* or "Bulimia Nervosa" or Bulimia* or "Eating Disorder*" or Rumination or "Restrict* Eat*" or "Atypical Purging" or "Disord* Eat*" or "Eating Path*" or "Night Eating*" or "Patholog* Eat*" or Pica or "Purging Disorder*").ti,ab. |
| 3 | 1 or 2 |
| 4 | exp "health care cost"/ or exp "cost of illness"/ or exp financial stress/ |
| 5 | ("Economic burden" or "Cost of illness" or "Cost of disease" or "Cost of sickness" or "Illness cost*" or "Economic cost*" or "Burden of illness" or "Burden of sickness" or "Financial burden" or "Medical cost*" or "Indirect cost*" or "Direct cost*").ti,ab. |
| 6 | ("economic study" or "econ* assess*" or "econ* conseq*" or "resource* use*" or "health* resource*" or "resource* utili*" or "health expendit*" or "out of pocket" or out-of-pocket or co-pay* or "time cost*" or "cost of caregiver*" or "Caregiver* cost" or "Societal cost*" or "Productivity cost*" or "Productivity loss*" or "Human capital cost*" or "Caregiv* burden" or "Carer* burden" or "Family burden").ti,ab. |
| 7 | (cost* adj3 (health* or ill* or disease* or time or society)).ti,ab. |
| 8 | (cost* adj2 (analys* or assess*OR study)).ti,ab. |
| 9 | ("Financial Stress" or "cost allocation" or health care cost*).ti,ab. |
| 10 | 4 or 5 or 6 or 7 or 8 or 9 |
| 11 | 3 and 10 |
| 12 | limit 11 to dc=20130701-20240630 [July 1st, 2013 to June 30th, 2024] |

Note: Search conducted on July 1, 2024

## Supplementary Table 3: Search strategy in PsycINFO (Ovid)

| 1 | exp eating disorders/ or exp Binge Eating/ |
| --- | --- |
| 2 | ("Abnormal Eat*" or Anorexia* or "Anorexia Nervosa" or "Avoidant Restrictive Food" or Binge* or "Bulimia Nervosa" or Bulimia* or "Eating Disorder*" or Rumination or "Restrict* Eat*" or "Atypical Purging" or "Disord* Eat*" or "Eating Path*" or "Night Eating*" or "Patholog* Eat*" or Pica or "Purging Disorder*").ti,ab. |
| 3 | 1 or 2 |
| 4 | exp Health Care Costs/ or exp "Costs and Cost Analysis"/ or exp Health Care Economics/ or Financial Strain/ |
| 5 | ("Economic burden" or "Cost of illness" or "Cost of disease" or "Cost of sickness" or "Illness cost*" or "Economic cost*" or "Burden of illness" or "Burden of sickness" or "Financial burden" or "Medical cost*" or "Indirect cost*" or "Direct cost*").ti,ab. |
| 6 | ("economic study" or "econ* assess*" or "econ* conseq*" or "resource* use*" or "health* resource*" or "resource* utili*" or "health expendit*" or "out of pocket" or out-of-pocket or co-pay* or "time cost*" or "cost of caregiver*" or "Caregiver* cost" or "Societal cost*" or "Productivity cost*" or "Productivity loss*" or "Human capital cost*" or "Caregiv* burden" or "Carer* burden" or "Family burden").ti,ab. |
| 7 | (cost* adj3 (health* or ill* or disease* or time or society)).ti,ab. |
| 8 | (cost* adj2 (analys* or assess*OR study)).ti,ab. |
| 9 | ("Financial Stress" or "cost allocation" or health care cost*).ti,ab. |
| 10 | 4 or 5 or 6 or 7 or 8 or 9 |
| 11 | 3 and 10 |
| 12 | limit 11 to ch=20130701-20240630 [July 1st, 2013 to June 30th, 2024] |
| 13 | limit 11 to up=20130701-20240630 [July 1st, 2013 to June 30th, 2024] |
| 14 | 12 or 13 |

Note: Search conducted on July 1, 2024

## Supplementary Table 4: Studies published in languages other than English and reasons for inclusion/exclusion

| **Author (year)** | **English title** | **Title in the main language** | **Journal** | **Language** | **English abstract available** | **Translated in Google** | **Included** | **Reason for exclusion** |
| --- | --- | --- | --- | --- | --- | --- | --- | --- |
| Bauer et al. (2013) (1) | Aftercare based on text messaging: Services across health care sectors for patients with bulimia nervosa | SMS-Nachsorge: Sektorenübergreifende Versorgung für Patientinnen mit Bulimia nervosa | Verhaltenstherapie | German | Yes | No | No | Not an eligible cost study |
| Mekui, C. A. and Weber, K. (2015) (2) | Eating disorders and psychiatric day hospital treatment | Troubles du comportement alimentaire et prise en charge en hôpital de jour psychiatrique | Revue Medicale Suisse | French | No | Yes | No | Not an eligible cost study |
| Peng et al. (2020) (3) | Reliability and validity of self-rated Family Burden Scale in families of the patients with anorexia nervosa | 自评家庭负担量表应用于神经性厌食患者家庭的信效度研究 | Journal of Shanghai Jiaotong University (Medical Science) | Chinese | Yes | No | No | Not an eligible cost study |

Note: In this systematic review, a total of 22 literature were found to be published in languages other than English. Out of these 22 studies, 3 studies were related to eating disorders. However, none of these studies were eligible economic cost studies for this systematic review.

## Supplementary Table 5: Excluded economic cost studies due to identical cost information available in other literature

| **Author (year)** | **Title of the excluded cost studies** | **Studies included** |
| --- | --- | --- |
| Author unknown (2015) (4) | How much does it cost to treat teens with anorexia nervosa in hospital? | Toulany et al. (2015) |
| Bellows et al. (2014) (5) | Health care costs of patients with binge eating disorder compared to patients with eating disorder not otherwise specified and no eating disorder | Bellows et al. (2015) |
| Deloitte Access Economics (2020) (6) | Social and economic cost of eating disorders in the United States of America | Streatfeild et al. et al. (2021) |
| Obeid et al. (2024) (7) | The financial and social impacts of the COVID‑19 pandemic on youth with eating disorders, their families, clinicians and the mental health system: a mixed methods cost analysis | Deloitte Access Economics (2023) |
| Stuhldreher et al. (2013) (8) | Cost-of-illness and its determinants in anorexia nervosa: Baseline results from the ANTOP study | Stuhldreher et al. (2015) |

**Additional references (Supplementary Tables 4 and 5)**

1. Bauer S, Okon E, Meermann R, Kordy H. SMS-Nachsorge: Sektorenübergreifende Versorgung für Patientinnen mit Bulimia nervosa [Aftercare based on text messaging: Services across health care sectors for patients with bulimia nervosa]. Verhaltenstherapie. 2013;23(3):204-9 doi: 10.1159/000354660

2. Mekui CA, Weber K. Troubles du comportement alimentaire et prise en charge en hôpital de jour psychiatrique [Eating disorders and psychiatric day hospital treatment]. Rev Med Suisse. 2015;11(461):406-8 doi: 10.53738/REVMED.2015.11.461.0406

3. Peng YH, Huang Y, Nie LY, Liu Q, Chen J. Reliability and validity of self-rated Family Burden Scale in families of the patients with anorexia nervosa. Journal of Shanghai Jiaotong University (Medical Science). 2020;40(6):804-8 doi: 10.3969/j.issn.1674-8115.2020.06.015

4. How much does it cost to treat teens with anorexia nervosa in hospital? Can Med Assoc J. 2015;187(9):658 doi: 10.1503/cmaj.150563

5. Bellows BK, Lafleur J, Kamauu A, Pawaskar M, Supina D, Babcock T, et al. Health care costs of patients with binge eating disorder compared to patients with eating disorder not otherwise specified and no eating disorder. Value Health. 2014;17(3) doi: 10.1016/j.jval.2014.03.1254

6. Deloitte Access Economics. (2020). The social and economic cost of eating disorders in the United States of America: A report for the Strategic Training Initiative for the Prevention of Eating Disorders and the Academy for Eating Disorders. Strategic Training Initiative for the Prevention of Eating Disorders and the Academy for Eating Disorders. [Accessed on 2024 Mar 01] Available from <https://www.hsph.harvard.edu/striped/report-economic-costs-of-eating-disorders/>

7. Obeid N, Coelho JS, Booij L, Dimitropoulos G, Silva-Roy P, Bartram M, et al. Estimating additional health and social costs in eating disorder care for young people during the COVID-19 pandemic: implications for surveillance and system transformation. Journal of eating disorders. 2024;12(1):52 doi: 10.1186/s40337-024-01003-1

8. Stuhldreher N, Konnopka A, König HH, ANTOP Study Group. Cost-of-illness and its determinants in anorexia nervosa: Baseline results from the ANTOP study. J Ment Health Policy Econ. 2013;16–S34.

## Supplementary Table 6: Annual total costs (in millions) associated with eating disorders

| **Type of ED** | **Author** | **Country** | **Year of costing** | **Currency** | **Health system cost** | **Direct Cost** | **Indirect Cost** | **Total cost** |
| --- | --- | --- | --- | --- | --- | --- | --- | --- |
| AN | Tannous et al. (2021) | Australia | 2018 | AUD | 51.0 | 52.7 | 100.7 | 153.4 |
|  | Gill et al. (2022) | Canada | 2018 | USD | 31.5 |  |  | 31.5 |
|  | Lee et al. (2021) | South Korea | 2015 | USD | 1.1 | 1.4 | 0.5 | 1.9 |
|  | Streatfeild et al. (2021) | USA | 2019 | USD | 1,067.1 | 1,067.1 | 10,095.4 | 11,162.3 |
|  | Marchili et al. (2024) | Italy | 2020-22 | EUR | 1.7 | 1.7 |  | 1.7 |
|  | Butterfly Foundation (2024) | Australia | 2022-23 | AUD | 188.1 | 188.1 | 840.7 | 1,028.8 |
| BN | Tannous et al. (2021) | Australia | 2018 | AUD | 122.8 | 198.8 | 176.9 | 375.7 |
|  | Lee et al. (2021) | South Korea | 2015 | USD | 0.8 | 0.9 | 0.4 | 1.2 |
|  | Streatfeild et al. (2021) | USA | 2019 | USD | 830.3 | 830.3 | 10,539.3 | 11,369.6 |
|  | Patel et al. (2018) | USA | 2014 | USD | 28.3 |  |  | 28.8 |
|  | Butterfly Foundation (2024) | Australia | 2022-23 | AUD | 20.5 | 251.3 | 2,530.2 | 2,781.5 |
| BED | Tannous et al. (2021) | Australia | 2018 | AUD | 72.9 | 139.1 | 131.1 | 270.1 |
|  | Jenkins (2022) | UK | 2017 | GBP |  |  |  | 3,470.0 |
|  | Streatfeild et al. (2021) | USA | 2019 | USD | 1,199.3 | 1,199.3 | 18,184.9 | 19,384.2 |
|  | Butterfly Foundation (2024) | Australia | 2022-23 | AUD | 10.6 | 252.8 | 4,745.6 | 4,998.1 |
| OSFED and UFED, or EDNOS | Tannous et al. (2021) | Australia | 2018 | AUD | 746.7 | 1,162.5 | 1,788.1 | 2,950.7 |
|  | Lee et al. (2021) | South Korea | 2015 | USD | 0.9 | 1.1 | 1.2 | 2.3 |
|  | Streatfeild et al. (2021) | USA | 2019 | USD | 1,458.8 | 1,458.8 | 21,340.8 | 22,799.6 |
|  | Butterfly Foundation (2024) | Australia | 2022-23 | AUD | 32.3 | 32.3 | 11883.3 | 11915.6 |
| EDs | Tannous et al. (2021) | Australia | 2018 | AUD | 993.4 | 1,553.2 | 2,196.7 | 3,749.9 |
|  | de Oliveira et al. (2016) | Canada | 2012 | CAD |  | 3.0 |  | 3.0 |
|  | de Oliveira et al. (2017) | Canada | 2012 | CAD | 63.0 |  |  | 63.0 |
|  | Lee et al. (2021) | South Korea | 2015 | USD | 2.8 | 3.4 | 2.1 | 5.5 |
|  | Streatfeild et al. (2021) | USA | 2019 | USD | 4,555.4 | 4,555.4 | 60,160.5 | 64,716.0 |
|  | Butterfly Foundation (2024) | Australia | 2022-23 | AUD | 241.4 | 785.4 | 19999.5 | 20,784.9 |
|  | Deloitte Access Economics (2023) | Canada | 2023 | CAD | 55.3-73.3 |  |  | 55.3-73.3 |

Note: EDs: eating disorders; AN: anorexia nervosa; BN: bulimia nervosa; BED: binge eating disorder; OSFED: other specified feeding and eating disorder; UFED: other unspecified feeding and eating disorder; EDNOS: eating disorders not otherwise specified

## Supplementary Table 7: Annual cost per-patient (in PPP-USD^+^) associated with eating disorders

| **Type of ED** | **Author** | **Country** | **Direct Cost** | **Indirect Cost** | **Total cost** | **Excess direct cost** | **Excess indirect cost** | **Excess total cost** |
| --- | --- | --- | --- | --- | --- | --- | --- | --- |
| AN | Butterfly foundation (2024) | Australia | 3,485 | 15,320 | 18,805 |  |  |  |
|  | Gatt et al. (2014) | Australia | 1,375 |  | 1,375 |  |  |  |
|  | Gill et al. (2022) | Canada | 22,575 |  | 22,575 |  |  |  |
|  | Toulany et al. (2015) | Canada | 55,716 | 7,775 | 63,491 |  |  |  |
|  | Stuhldreher et al. (2015) | Germany | 25,627 | 18,928 | 44,555 |  |  |  |
|  | Bothe et al. (2021) | Germany |  |  | 23,612 |  |  |  |
|  | Tseng et al. (2021) | Taiwan |  |  |  | 910 |  | 910 |
|  | Ballard and Crane (2015) | USA | 643 |  |  |  |  |  |
|  | Streatfeild et al. (2021) | USA | 2,848 | 26,945 | 29,792 |  |  |  |
|  | Marchili et al. (2024) | Italy | 9584 |  | 9,584 |  |  |  |
| BN | Butterfly Foundation (2024) | Australia | 1,414 | 14,235 | 15,649 |  |  |  |
|  | Gatt et al. (2014) | Australia | 2,862 |  | 2,862 |  |  |  |
|  | Bothe et al. (2021) | Germany |  |  | 60,896 |  |  |  |
|  | Tseng et al. (2021) | Taiwan |  |  |  | 368 |  | 368 |
|  | Jenkins (2022) | UK | 883 | 5,326 | 5,838 |  |  |  |
|  | Ballard and Crane (2015) | USA | 534 |  |  |  |  |  |
|  | Patel et al. (2018) | USA | 40,588 |  | 40,588 |  |  |  |
|  | Streatfeild et al. (2021) | USA | 1,454 | 18,455 | 19,909 |  |  |  |
| BED | Butterfly Foundation (2024) | Australia | 762 | 14,307 | 15,069 |  |  |  |
|  | Jenkins (2022) | UK | 640 | 2,883 | 3,517 |  |  |  |
|  | Jenkins (2022) | UK | 795 | 4,851 | 5,361 |  |  |  |
|  | Bellows et al. (2015) | USA | 42,008 |  | 42,008 |  |  |  |
|  | Ling et al. (2017) | USA | 20,194 | 19,327 | 35,519 | 5,729 | 10,295 | 16,024 |
|  | Streatfeild et al. (2021) | USA | 642 | 9,738 | 10,381 |  |  |  |
| OSFED and UFED, or EDNOS | Butterfly Foundation (2024) | Australia | 90 | 11,891 | 11,981 |  |  |  |
|  | Jenkins (2022) | UK | 686 | 5,521 | 6,072 |  |  |  |
|  | Bellows et al. (2015) | USA | 46,165 |  | 46,165 |  |  |  |
|  | Streatfeild et al. (2021) | USA | 657 | 9,613 | 10,270 |  |  |  |
|  | Ballard and Crane (2015) | USA | 415 |  |  |  |  |  |
| EDs | Butterfly Foundation (2024) | Australia | 502 | 12,790 | 13,292 |  |  |  |
|  | Tannous et al. (2021) | Australia | 6,302 | 14,396 | 20,698 |  |  |  |
|  | de Oliveira et al. (2017) | Canada |  |  |  | 8,434 |  | 8,434 |
|  | de Oliveira et al. (2023) | Canada | 7,124 |  | 7,124 |  |  |  |
|  | Kurisu et al. (2023) | Japan | 862 |  | 862 |  |  |  |
|  | Samnaliev et al. (2015) | USA | 7,200 | 34,611 | 41,811 | 2,329 | 2,608 | 4,936 |
|  | Streatfeild et al. (2021) | USA | 905 | 11,953 | 12,858 |  |  |  |
|  | Presskreischer et al. (2022) | USA | 34,071 |  | 34,071 | 25,491 |  | 25,491 |
|  | Surgenor et al. (2022) | New Zealand | 5,918 | 9,349 | 15,267 |  |  |  |

Notes: ED: eating disorder; AN: anorexia nervosa; BN: bulimia nervosa; BED: binge eating disorder; OSFED: other specified feeding and eating disorder; UFED: other unspecified feeding and eating disorder; EDNOS: eating disorders not otherwise specified

^+^ All cost estimates were converted into 2024 USD using gross domestic product (GDP) deflator and relevant countries' purchasing power parity (PPP) exchange rates.

## Supplementary Table 8: Annual cost per-patient associated with eating disorders

| **Type of ED** | **Author** | **Country** | **Year of costing** | **Currency** | **Direct Cost** | **Indirect Cost** | **Total cost** | **Excess direct cost** | **Excess indirect cost** | **Excess total cost** |
| --- | --- | --- | --- | --- | --- | --- | --- | --- | --- | --- |
| AN | Gatt et al. (2014) | Australia | 2012 | AUD | 1,525 |  | 1,525 |  |  |  |
|  | Gill et al. (2022) | Canada | 2018 | USD | 18,398 |  | 18,398 |  |  |  |
|  | Toulany et al. (2015) | Canada | 2013 | CAD | 51,349 | 7,166 | 58,515 |  |  |  |
|  | Stuhldreher et al. (2015) | Germany | 2008 | EUR | 13,496 | 9,968 | 23,464 |  |  |  |
|  | Bothe et al. (2021) | Germany | 2018 | EUR |  |  | 14,249 |  |  |  |
|  | Tseng et al. (2021) | Taiwan | 2013 | USD |  |  |  | 792 |  | 792 |
|  | Streatfeild et al. (2021) | USA | 2019 | USD | 2,615 | 24,744 | 27,359 |  |  |  |
|  | Ballard and Crane (2015) | USA | 2006 | USD | 474 |  |  |  |  |  |
|  | Marchili et al. (2024) | Italy | 2020-22 | EUR | 6,447 |  | 6,447 |  |  |  |
| BN | Butterfly Foundation (2024) | Australia | 2022-23 | AUD | 4,941 | 21,719 | 26,660 |  |  |  |
|  | Gatt et al. (2014) | Australia | 2012 | AUD | 3,175 |  | 3,175 |  |  |  |
|  | Bothe et al. (2021) | Germany | 2018 | EUR |  |  | 36,749 |  |  |  |
|  | Tseng et al. (2021) | Taiwan | 2013 | USD |  |  |  | 320 |  | 320 |
|  | Jenkins (2022) | UK | 2017 | GBP | 538 | 3,247 | 3,559 |  |  |  |
|  | Streatfeild et al. (2021) | USA | 2019 | USD | 1,335 | 16,948 | 18,283 |  |  |  |
|  | Patel et al. (2018) | USA | 2014 | USD | 34,398 |  | 34,398 |  |  |  |
|  | Ballard and Crane (2015) | USA | 2006 | USD | 394 |  |  |  |  |  |
| BED | Butterfly Foundation (2024) | Australia | 2022-23 | AUD | 2,004 | 20,181 | 22,185 |  |  |  |
|  | Jenkins (2022) | UK | 2017 | GBP | 485 | 2,958 | 3,268 |  |  |  |
|  | Jenkins (2022) | UK | 2017 | GBP | 390 | 1,758 | 2,144 |  |  |  |
|  | Ling et al. (2017) | USA | 2013 | USD | 20,194 | 19,327 | 35,519 | 5,729 | 10,295 | 16,024 |
|  | Streatfeild et al. (2021) | USA | 2019 | USD | 590 | 8,943 | 9,533 |  |  |  |
|  | Bellows et al. (2015) | USA | 2011 | USD | 33,716 |  | 33,716 |  |  |  |
| OSFED and UFED, or EDNOS | Butterfly Foundation (2024) | Australia | 2022-23 | AUD | 1,080 | 20,283 | 21,363 |  |  |  |
|  | Jenkins (2022) | UK | 2017 | GBP | 418 | 3,366 | 3,702 |  |  |  |
|  | Bellows et al. (2015) | USA | 2011 | USD | 37,052 |  | 37,052 |  |  |  |
|  | Streatfeild et al. (2021) | USA | 2019 | USD | 603 | 8,828 | 9,431 |  |  |  |
|  | Ballard and Crane (2015) | USA | 2006 | USD | 306 |  |  |  |  |  |
| EDs | Butterfly Foundation (2024) | Australia | 2022-23 | AUD | 128 | 16,857 | 16,985 |  |  |  |
|  | Tannous et al. (2021) | Australia | 2018 | AUD | 7,541 | 17,228 | 24,769 |  |  |  |
|  | de Oliveira et al. (2023) | Canada | 2021 | CAD | 7,700 |  | 7,700 |  |  |  |
|  | de Oliveira et al. (2017) | Canada | 2012 | CAD |  |  |  | 7,640 |  | 7,640 |
|  | Kurisu et al. (2023) | Japan | 2017 | USD | 794 |  | 794 |  |  |  |
|  | Presskreischer et al. (2022) | USA | 2016 | USD | 29,456 |  | 29,456 | 22,038 |  | 22,038 |
|  | Samnaliev et al. (2015) | USA | 2011 | USD | 5,779 | 27,779 | 33,558 | 1,869 | 2,093 | 3,962 |
|  | Streatfeild et al. (2021) | USA | 2019 | USD | 831 | 10,977 | 11,808 |  |  |  |
|  | Surgenor et al. (2022) | New Zealand | 2020 | NZD | 7,375 | 11,650 | 19,025 |  |  |  |
|  | Butterfly Foundation (2024) | Australia | 2022-23 | AUD | 712 | 18,132 | 18,844 |  |  |  |

Notes: EDs: eating disorders; AN: anorexia nervosa; BN: bulimia nervosa; BED: binge eating disorder; OSFED: other specified feeding and eating disorder; UFED: other unspecified feeding and eating disorder; EDNOS: eating disorders not otherwise specified
